# Emerging investigator series: Coprecipitation with glucuronic acid limits reductive dissolution and transformation of ferrihydrite in an anoxic soil†

**DOI:** 10.1039/d4em00238e

**Published:** 2024-07-25

**Authors:** Laurel K. ThomasArrigo, Luiza Notini, Sophie Vontobel, Sylvain Bouchet, Tabea Nydegger, Ruben Kretzschmar

**Affiliations:** a Environmental Chemistry Group, Institute of Chemistry, University of Neuchâtel Avenue de Bellevaux 51 CH-2000 Neuchâtel Switzerland laurel.thomas@unine.ch; b Soil Chemistry Group, Institute of Biogeochemistry and Pollutant Dynamics, Department of Environmental Systems Science, ETH Zurich Universitätstrasse 16, CHN CH-8092 Zurich Switzerland

## Abstract

Ferrihydrite, a poorly crystalline Fe(iii)-oxyhydroxide, is abundant in soils and is often found associated with organic matter. Model studies consistently show that in the presence of aqueous Fe(ii), organic carbon (OC)-associated ferrihydrite undergoes less transformation than OC-free ferrihydrite. Yet, these findings contrast microbial reductive dissolution studies in which the OC promotes the reductive dissolution of Fe(iii) in ferrihydrite and leads to the release of associated OC. To shed light on these complex processes, we quantified the extent of reductive dissolution and transformation of native Fe minerals and added ferrihydrite in anoxic soil incubations where pure ^57^Fe-ferrihydrite (^57^Fh), pure ^57^Fe-ferrihydrite plus dissolved glucuronic acid (^57^Fh + GluC_aq_), a ^57^Fe-ferrihydrite-^13^C-glucuronic acid coprecipitate (^57^Fh^13^GluC), or only dissolved glucuronic acid (^13^GluC_aq_) were added. By tracking the transformation of the ^57^Fe-ferrihydrite in the solid phase with Mössbauer spectroscopy together with analysis of the iron isotope composition of the aqueous phase and chemical extractions with inductively coupled plasma-mass spectrometry, we show that the pure ^57^Fe-ferrihydrite underwent more reductive dissolution and transformation than the coprecipitated ^57^Fe-ferrihydrite when identical amounts of glucuronic acid were provided (^57^Fh + GluC_aq_*versus*^57^Fh^13^GluC treatments). In the absence of glucuronic acid, the pure ^57^Fe-ferrihydrite underwent the least reductive dissolution and transformation (^57^Fh). Comparing all treatments, the overall extent of Fe(iii) reduction, including the added and native Fe minerals, determined with X-ray absorption spectroscopy, was highest in the ^57^Fh + GluC_aq_ treatment. Collectively, our results suggest that the limited bioavailability of the coprecipitated OC restricts not only the reductive dissolution of the coprecipitated mineral, but it also limits the enhanced reduction of native soil Fe(iii) minerals.

## Abstract 2

Ferrihydrite, a poorly crystalline Fe(iii)-oxyhydroxide, is abundant in soils and is often found associated with organic matter.

## Graphical abstract

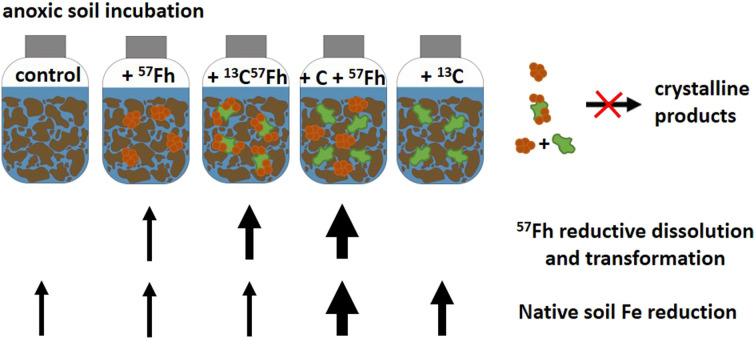

**Environmental significance**

Coprecipitation of organic carbon with ferrihydrite limits its bioavailability as a carbon source to microorganisms. Under sub- or anoxic conditions, reductive dissolution and transformation of ferrihydrite may lead to the release of adsorbed organic carbon. Yet, this process is concomitantly influenced by the organic carbon itself. Here, we utilize stable iron isotopes to follow the fate of ferrihydrite in an anoxic soil incubation. We demonstrate that ferrihydrite coprecipitated with organic carbon undergoes less reductive dissolution and transformation compared to pure ferrihydrite with the same amount of organic carbon added separately. The coprecipitated organic carbon also led to less reduction of Fe in native soil minerals than the same amount of organic carbon added in dissolved form.

## Introduction

Ferrihydrite is a poorly crystalline Fe(iii)-oxyhydroxide commonly formed at redox interfaces in soils and sediments, where oxidation of ferrous iron (Fe(ii)) to ferric Fe (Fe(iii)) and its rapid hydrolysis leads to the precipitation of this short-range ordered (SRO) iron mineral. With a high specific surface area and point of zero charge (PZC) near pH 8,^1^ ferrihydrite is often linked to the sorption of high amounts of soil organic matter (SOM) and thus contributes to the stabilization of soil organic carbon (OC) *via* mineral protection mechanisms, particularly in well-aerated soils.^2,3^ However, under anoxic conditions, Fe(iii) can be used as the terminal electron acceptor during anaerobic respiration of OC.^4–7^ In soils, the reductive dissolution of Fe(iii)-(oxyhydr)oxides leads to the release of mineral-associated OC to soil solution^8–13^ as dissolved organic carbon (DOC)^14–17^ or in organic-Fe/Al colloids.^18–20^ Because SRO-iron minerals have a high propensity for rapid reductive dissolution,^21^ ferrihydrite likely plays an active role in the biogeochemical cycling of C, particularly in redox active soils and sediments.

Dissimilatory reductive dissolution of Fe(iii)-(oxyhydr)oxides leads to the formation of more crystalline mineral phases, driven, in part, by exposure to aqueous Fe(ii). Adsorption of Fe(ii) onto Fe(iii)-(oxyhydr)oxide surfaces results in the oxidation of the Fe(ii) and the transfer of electrons to structural Fe(iii), which is subsequently released to solution as Fe(ii). Such electron transfer processes trigger mineral recrystallization^22,23^ or transformation.^24–26^ For ferrihydrite, a thermodynamically unstable mineral phase, reductive dissolution and electron transfer processes lead to the formation of more crystalline mineral products. Model studies involving additions of aqueous Fe(ii) show that depending on Fe(ii) concentrations, pH, and background ligands, resulting minerals can include lepidocrocite, goethite, and magnetite.^24,25,27,28^ Model studies following microbial reductive dissolution of ferrihydrite similarly report crystalline products, including magnetite, chukanovite, goethite and green rust.^29,30^

The kinetics of reductive dissolution of ferrihydrite and the pathways of ferrihydrite transformations are influenced by the presence of adsorbed, coprecipitated, or dissolved OC. Adsorbed OC reduces the number of available sorption sites on the mineral surface and coprecipitation with OC changes the particle size, morphology, PZC, and aggregate size and density of the resulting mineral.^1,31–35^ In model studies involving additions of aqueous Fe(ii), ferrihydrite with adsorbed or coprecipitated OC consistently shows less mineral transformation than OC-free ferrihydrite^1,36–41^ despite similar range of removal of Fe(ii) from aqueous solution,^40^ favoring instead recrystallization and the formation of thin lepidocrocite lamina over crystalline products.^1,40^ These findings suggest that ferrihydrite coprecipitated with OC may remain an efficient sorbent phase for OC even in the presence of aqueous Fe(ii). Yet, this contrasts results from microbial reductive dissolution studies, where adsorbed or coprecipitated OC facilitates an increased mineral reactivity as various Fe(iii)-reducing microorganisms can utilize quinone moieties either in the adsorbed or coprecipitated OC or can produce endogenous electron-shuttling or chelating compounds, resulting in faster Fe(iii) reduction rates in coprecipitates compared to the OC-free ferrihydrite.^10,12,42–44^

In soils, ferrihydrite often forms in the presence of dissolved or particulate OC, resulting in mineral–organic associations.^5,45–53^ Because of the important role that ferrihydrite plays for C cycling in soils, it is critical to understand the factors driving the stabilization, recrystallization, or transformation of ferrihydrite in soils, and to clarify the role of mineral-associated OC as a facilitator or inhibitor of these processes. In a previous study, we used isotope labelling (^13^C and ^57^Fe) of a low molecular weight organic acid (LMWOa; glucuronic acid) and ferrihydrite to demonstrate that coprecipitation with ferrihydrite significantly limited the bioavailability of the glucuronic acid in an anoxic soil incubation.^17^ In the current study, we build on these results, incorporating the recently developed application of isotope labeling of minerals in combination with ^57^Fe Mössbauer spectroscopy^54^ to assess the extent to which coprecipitation with glucuronic acid alters the microbial availability of Fe(iii) in a pure ^57^Fe-labelled ferrihydrite *versus* a ^57^Fe-labelled ferrihydrite-^13^C-glucuronic acid coprecipitate in an anoxic soil incubation. Because the addition of the glucuronic acid was previously found to strongly stimulate the soil microbial activity,^17^ we also included here a treatment in which ^57^Fe-labelled ferrihydrite and glucuronic acid were added in simultaneous but separate spikes. To compliment the results of Mössbauer spectroscopy, the fate of Fe(iii) in the ^57^Fe-labelled ferrihydrites was additionally followed through time-resolved analysis of the iron isotope composition of the aqueous phase and in selective chemical extractions targeting adsorbed Fe(ii) (0.5 M HCl), poorly crystalline or amorphous Fe (acid ammonium oxalate), and organically-complexed or colloidal Fe (Na-pyrophosphate). Collectively, this approach enabled a wholistic understanding of the dynamic transformation of Fe(iii) in ferrihydrite during reductive dissolution in the presence of OC and a natural soil matrix. To understand the overall progress of Fe(iii) reduction in the system, aqueous geochemical parameters were monitored and changes in iron oxidation state and speciation in the bulk soils were analyzed with X-ray absorption spectroscopy. The results of this study highlight the intricate coupling of biogeochemical cycles of iron and carbon in anoxic soils.

## Materials and methods

### Study site

Ferrihydrite is a common mineral phase found in mineral- and organic-rich soils of Iceland.^20,55^ For this study, a subsoil horizon (60–72 cm depth) was collected in July 2020, from the Hestur_GA soil profile;^17,20,53^ a ferrihydrite-containing^20^ Gleyic Andosol (GA; Icelandic soil classification system) representative of the drainage-impacted wetlands typical across northern and western Iceland.^55^ The field site is located in the Borgarfjörður catchment in western Iceland (Fig. S1†). Basalts in this region are primarily tertiary (older than 3.1 Ma)^56^ and the region receives a low influx of aeolian deposition (25–100 g m^−2^ per years).^57^ Additional details to the study site, a description of soil sampling and characterization of the soil horizons have been previously published^17^ and are found in detail in the ESI.† Briefly, for the soil collected in 2020, soil pH_H_2_O_ was 4.56 and total Fe and C contents were 73.1 mg g^−1^ and 21.6 wt%, respectively. X-ray diffraction patterns indicated the presence of plagioclase feldspars, pyroxenes, small contributions from quartz and a significant amorphous fraction.^17^

### Soil slurry incubation set-up and sampling

All solutions used in this experiment were prepared from ultrapure water (UPW, Milli-Q®, Millipore, >18.2 MΩ cm). A description of the synthesis of isotope-labelled ferrihydrite (^57^Fh) and the ferrihydrite-glucuronic acid coprecipitate (^57^Fh^13^GluC, C : Fe molar ratio = 0.42) using ^57^Fe-labelled Fe(0) metal powder and ^13^C-labelled glucuronic acid and a detailed description of the resulting (co-)precipitates have been previously published^17^ and are therefore found in the ESI.†

Prior to starting the experiment, the field-moist soils were sieved to <2 mm with a nylon sieve and visible plant or root material was removed with tweezers. The prepared soils were then packaged into plastic bags and kept at 25 °C in the dark for two weeks to allow soil microorganisms to recover from 4 °C storage. Soil incubations were performed in triplicate as soil slurries (soil : water ratio of 1 : 10) in Al-wrapped septum bottles. Five treatments were considered: soil amended with ^57^Fh (^57^Fh), soil amended with ^13^GluC (^13^GluC_aq_), soil amended with the ^57^Fh^13^GluC coprecipitate (^57^Fh^13^GluC), soil amended with ^57^Fh and a non-isotope labelled (natural isotope abundance) GluC (d-glucuronic acid, Sigma) added simultaneously but separately (^57^Fh + GluC_aq_), and a control which received no amendments (Control, Table S2†). The soil (6.82 g, equivalent to 3.5 of dry soil, respectively) was added to 117 mL septum bottles which were then moved into an anoxic glovebox (MBRAUN, N_2_ atmosphere, <1 ppm (v/v) O_2_) and covered in Parafilm (to allow gas exchange but prevent evapotranspiration). After 24 hours, 30.2 mL of anoxic UPW (or 28.7 mL for the ^57^Fh + GluC_aq_ treatment) was added followed immediately by the amendment spikes. To this end, the synthesized coprecipitates ^57^Fh and ^57^Fh^13^GluC or ^(13−)^GluC were resuspended in 1.5 mL of anoxic UPW directly prior to spiking to the septum bottles. The control treatment similarly received 1.5 mL of UPW. Immediately following the amendment spikes, the bottles were crimp-sealed with rubber stoppers and removed from the glovebox. All treatment bottles were then placed on an orbital shaker (150 rpm) in a temperature-controlled room at 25 °C. After 72 h and 1, 2, 4, 5, and 6 weeks, the septum bottles were moved into the glovebox, where they were opened for anoxic measurements of pH and Eh and sampling of the aqueous and solid phase. Details to the sampling procedure have been previously published^17^ and thus are provided here in the ESI.†

### Aqueous-phase analyses

Filtered aqueous samples and the selective chemical extractions (described below) were measured for Fe concentration with inductively coupled plasma-optical emission spectrometry (ICP-OES, Agilent 5100). The Fe isotope composition of the aqueous phase and the chemical extracts was measured by inductively coupled plasma mass spectrometry (ICP-MS, Agilent 8800 Triple Quad) as previously described.^1,40^ Iron isotope composition results are reported as fractions (*f*) of the isotope *n* (*f*^*n*^Fe), whereby the counts per second (cps) of the isotope of interest *n* is divided by the sum cps of the Fe isotopes ^56^Fe and ^57^Fe (for aqueous Fe samples) and ^54^Fe, ^56^Fe, ^57^Fe and ^58^Fe for the chemical extractions. Dissolved organic carbon (DOC) in the 0.22 μm aqueous-phase filtrates was measured with a Dimatoc 2000 TOC analyzer (Dimatec).

### Selective chemical extractions

Selective chemical extractions were used to estimate the amount of Fe in various chemical forms and its isotope composition in the initial soil and at selected timepoints during the soil incubation. We used a two-step extraction procedure. First, homogenized samples were subjected to 0.5 M HCl extraction (2 h on a horizontal shaker (150 rpm) in the glovebox in the dark). The extracts were then centrifuged (18 620 RCF for 10 min) and the supernatant carefully pipetted off. This 0.5 M HCl-extractable fraction comprises the majority of adsorbed Fe(ii) and surface Fe-oxyhydroxide layers in soils^58^ in addition to poorly-crystalline minerals like ferrihydrite.^42^ The amount of adsorbed Fe(ii) was then estimated by measuring Fe(ii) in the extracts with the 1,10-phenanthroline method.^59,60^ The remaining pellet was then re-suspended in an acid ammonium oxalate solution (pH = 3, 0.2 M ammonium oxalate in 4 : 3 ratio with oxalic acid) for 4 hours (horizontal shaker, 150 rpm), prior to centrifuging and collecting of the supernatant as described above in order to estimate the amount of Fe in poorly-crystalline or amorphous mineral form (‘Fe_o_’).^59^ No extractions were included to target Fe in crystalline clay minerals (*e.g.* illite, kaolinite, smectite). Recently, smectite was identified in select horizons of Icelandic Histosols.^61^ However, in general, Icelandic Andosols lack considerable amounts of crystalline phyllosilicates, with the rapid weathering of volcanic material resulting instead in an abundance of short-range ordered aluminosilicates like allophane and imogolite.^55^ In agreement, crystalline phyllosilicates were not identified in bulk powder X-ray diffraction patterns of the soil used here.^17^ The ammonium oxalate solution was also chosen because previous work showed that, in Icelandic soils, the amount of Fe mobilized with ammonium oxalate is often higher than that mobilized in dithionite-citrate extractions, which target the total reactive Fe not bound in silicates.^20,61–63^ This suggests that a high amount of Fe is found in poorly-crystalline mineral phases, some of which may be incorporated into the structure of poorly crystalline aluminosilicates or occluded within allophane aggregate structures.^20^ Because anoxic incubation of similar Icelandic wetland soils resulted in the release of iron in a fine colloidal fraction (<3 kD to 0.45 μm size),^20^ we also estimated the amount of ‘organically-bound or colloidal Fe’ (Na-pyrophosphate;^64^ ‘Fe_p_’). To this end, a separate soil sample was treated with 0.1 M Na-pyrophosphate solution (pH = 10, 16 h, end-over-end shaking) and the supernatant was collected after centrifugation (3000*g*, 30 min). For the ‘Fe_p_’ treatment, triplicates of the solid-phase samples were manually homogenized and the treatment was performed in duplicate.

Collectively, the chosen extractions are expected to capture all possible fates of mobilized ^(57)^Fe, thus enabling a reasonable comparison between the isotope composition of the extractions and Mössbauer spectroscopy. Iron concentrations and the iron isotope composition in the acid ammonium oxalate extracts were corrected for the small amounts of Fe remaining in the soil pellet from the previous extract solution. Fractionation of iron isotopes may occur during ligand-controlled and reductive dissolution of Fe(iii) by oxalate, however the resulting enrichment factors (+0.5 to −2.6‰)^65^ are insignificant compared to the values obtained in this study. Total element concentrations and measurements of the Fe isotope composition in all the centrifuged extracts were conducted as described above.

### X-ray absorption spectroscopy

To follow changes in speciation of bulk solid-phase Fe, the (co-)precipitates (^57^Fe and ^57^Fh^13^GluC), the initial soil, and the incubated soil treatments were analyzed by bulk Fe K-edge (7112 eV) X-ray absorption spectroscopy (XAS) at the XAFS beamline of ELETTRA (Trieste, Italy) or at BM23 of ESRF (Grenoble, Italy). For these measurements, dried (un)reacted soil material (in triplicate) was manually homogenized with a mortar and pestle until all material passed a <350 μm sieve and then pressed into 10 mm pellets and sealed with Kapton® tape. At ELETTRA, X-ray absorption near edge structure (XANES) and extended X-ray absorption fine structure (EXAFS) spectra were recorded in transmission mode at ∼80 K using a N_2_(l) cryostat. At ESRF, spectra were recorded in transmission mode at ∼10 K using a He(l) cryostat. Additional details to XAS spectra collection and data analysis are found in the ESI.†

### Mössbauer spectroscopy

To follow the fate of iron added as ferrihydrite (^57^Fh) or organic-associated ferrihydrite (^57^Fh^13^GluC) in the solid-phase, ^57^Fe Mössbauer spectra were collected of the (co-)precipitates (^57^Fh and ^57^Fh^13^GluC), the initial soil, the mixtures of the (co-)precipitate and the initial soil (^57^Fh + soil and ^57^Fh^13^GluC + soil) and the 2- and 6 weeks incubated samples (^57^Fh, ^57^Fh^13^GluC, and ^57^Fh + GluC_aq_ treatments). Spectra were obtained using a ^57^Co/Rh γ-radiation source with an activity of ∼50 mCi vibrated in a constant acceleration mode in a standard setup (WissEl, Wissenschaftliche Elektronik GmbH). Dried sample material (50–150 mg) was sealed between two layers of Kapton® tape within a plastic circular frame (10 mm). Reacted samples were prepared under anoxic conditions, and triplicates of the solid-phase samples were manually homogenized to form a single sample. All samples were mounted in transmission geometry. Sample temperatures were maintained with a closed-cycle cryostat (SHI-850-5, Janis Research Co.), whereas the ^57^Co/Rh source remained at room temperature. Spectra were collected at 77, 45, 35, 25, 15, and 5 K and analyzed using the Recoil software (University of Ottawa, Canada) by applying an extended Voigt-based fitting routine.^66^ The spectra were calibrated against 7 μm thick α-^57^Fe(0) at 295 K, and center shifts (CS) are quoted relative to this. For all samples, the half width at half-maximum was fixed to 0.135 mm s^−1^; the value of the inner line broadening of the calibration foil at 295 K.

## Results and discussion

### Aqueous geochemical analyses

Trends in aqueous geochemistry in the soil slurries, including pH, Eh, dissolved Fe (Fe_aq_) and DOC, have been extensively discussed in the context of mineralization of the native SOM and the added glucuronic acid for the control, ^57^Fh, ^57^Fh^13^GluC, and ^13^GluC_aq_ treatments in ref. 17 and are summarized in Table 1. Briefly, aqueous geochemical data suggested that the addition of the readily available glucuronic acid in the ^13^GluC_aq_ treatment stimulated microbial activity, leading to the highest amount of CO_2_ produced, lowest Eh_7_ (the redox potential converted to pH 7), high pH, and high concentrations of Fe_aq_ and DOC measured in the porewater (Table 1, Fig. S3†).^17^ In contrast, coprecipitation with ferrihydrite reduced the bioavailability and thus the extent of mineralization of the glucuronic acid in the ^57^Fh^13^GluC treatment (Table 1).^17^ The addition of ferrihydrite in the ^57^Fh treatment resulted in similar CO_2_ production and aqueous geochemical trends similar to (Eh_7_, Fe_aq_) or lower than (pH, DOC) those observed in the control treatment, suggesting that the added ferrihydrite served primarily as a sorbent phase, removing otherwise released DOC from solution.^17^

**Table 1:** Aqueous geochemical parameters measured after 6 weeks of anoxic incubation of the soil slurries and cumulated CO_2_ and fraction of the added ^13^C-glucuronic acid mineralized. Time-resolved data is presented in Fig. S3 and ref. 17a

Sample	pH (−)	Eh_7_ (mV)	Fe_aq_ (mM)	DOC (mg L^−1^)	Total CO_2_ (mmol C per g soil)	SOM-derived CO_2_ (mmol C per g soil)	Fraction of ^13^C-glucuronic acid mineralized (%)
Control	5.96 (0.04)	45 (5)	1.44 (0.23)	174 (9)	0.071 (0.004)	0.071 (0.004)	—
^57^Fh	5.70 (0.06)	49 (7)	1.43 (0.19)	120 (3)	0.066 (0.003)	0.065 (0.003)	—
^57^Fh^13^GluC	6.35 (0.03)	−90 (29)	1.98 (0.16)	268 (41)	0.134 (0.006)	0.098 (0.004)	37.3 (2.9)
^57^Fh + GluC_aq_	6.94 (0.04)	−70 (3)	1.93 (0.06)	287 (13)	nm	nm	nm
^13^GluC_aq_	6.70 (0.05)	−112 (12)	2.13 (0.12)	400 (40)	0.172 (0.01)	0.122 (0.005)	51.5 (6.8)

a Errors in parentheses indicate the standard deviation between triplicate incubation bottles. Abbreviations: nm = not measured.

In general, trends in aqueous geochemical conditions in the ^57^Fh + GluC_aq_ treatment followed most closely those recorded in the ^13^GluC_aq_ treatment, with some noticeable differences (Fig. S3†). Firstly, the pH was slightly higher in the ^57^Fh + GluC_aq_ treatment. In anoxic soils, increases in pH are linked to the consumption of protons during the reductive dissolution of Fe(iii).^67^ Thus, the higher pH may indicate more Fe(iii) reduction, in agreement with slightly higher Fe_aq_ recorded in this treatment. Thus, it appears that the glucuronic acid added in the ^57^Fh + GluC_aq_ treatment was also readily available and stimulated the soil microbial community, despite the presence of additional ferrihydrite. Yet, DOC concentrations in the ^57^Fh + GluC_aq_ treatment were slightly lower than in the ^13^GluC_aq_ treatment, suggesting that the added ferrihydrite removed DOC from solution, akin to the role of the added ferrihydrite as a sorbent phase in the ^57^Fh treatment.^17^

### Bulk iron (mineral) dynamics

Changes in the oxidation state of solid-phase Fe, assessed by linear combination fitting of Fe K-edge XANES spectra, are shown in Fig. S4 and detailed in Table S3.† As expected, for all treatments, anoxic incubation led to increases in the fraction of solid-phase Fe(ii), which increased from ∼6% (of solid-phase Fe) in the initial soil to 13–16% in the control, ^57^Fh, and ^57^Fh^13^GluC treatments, to 25% in the ^13^GluC_aq_ treatment, and to 56% after 6 weeks in the ^57^Fh + GluC_aq_ treatment. Speciation changes in solid-phase Fe, assessed by linear combination fitting of Fe K-edge EXAFS spectra, are shown in Fig. 2 and detailed in Table 2. Initially, Fe in the soil comprised 41% ferrihydrite, 20% goethite, 29% Fe(ii)/(iii) in clay minerals, and 10% organically-complexed Fe(iii) (Table 2). After 6 weeks anoxic incubation, the fraction of ferrihydrite remained relatively high, with ∼38% of solid-phase Fe comprising ferrihydrite in the control and ^13^GluC_aq_ treatments. The addition of the ^57^Fe-labelled ferrihydrite to the ^57^Fh, ^57^Fh^13^GluC, and ^57^Fh + GluC_aq_ treatments increased the fraction of ferrihydrite initially present in these soils (Table 2), yet changes in the contribution of ferrihydrite to solid-phase Fe after 6 weeks anoxic incubation in the ^57^Fh and ^57^Fh^13^GluC were also small (from 50% to ∼47%). The fractions of Fe fit as goethite (19 ± 3%), Fe in clay minerals (27 ± 4%), and organically-complexed Fe(iii) (9 ± 1%) were similarly stable for all of the treatments. The most noticeable changes were seen in the ^57^Fh + GluC_aq_ treatment, where the contribution of ferrihydrite decreased from 50% to 20%, seemingly at the expense of the formation of organically-complexed Fe(ii) (24% of solid-phase Fe). A minor fraction of organically-complexed Fe(ii) (11% of solid-phase Fe) was recorded in the ^13^GluC_aq_ treatment as well. Despite the quality of the data and their respective fits (Fig. 2, NSSR <3.2%, Table 2) a slight systematic discrepancy exists between fractions of Fe(ii) and Fe(iii) determined from LCF analyses of XANES and EXAFS spectra (Table S5†). As this is only noticed for the 6 weeks incubated samples, it may suggest a missing Fe(ii) reference for the fitting the EXAFS spectra.

**Table 2:** Linear combination fit results for Fe K-edge EXAFS spectra of the initial soil and 6 weeks anoxic incubated treatments

Sample	Fh (%)	Gt (%)	Fe in clays^d^ (%)	Fe(iii)-organic (%)	Fe(ii)-organic (%)	NSSR^a^ (%)	red.^b^*χ*^2^ (−)
Initial soil	41	20	29	10		2.03	0.0911
Initial soil + ^57^Fh treatments^c^	50	17	25	9			
Control	37	23	32	8		2.26	0.0817
^57^Fh	48	18	27	8		2.43	0.0816
^57^Fh^13^GluC	47	17	26	10		2.43	0.0775
^57^Fh + GluC_aq_	20	21	26	9	24	3.16	0.0753
^13^GluC_aq_	39	15	23	11	11	2.65	0.0796

a NSSR: normalized sum of squared residuals (100 × ∑_i_(data_i_ − fit_i_)^2^/∑_i_data^2^).

b Fit accuracy (reduced *χ*^2^ = (*N*_idp_/*N*_pts_)∑_*i*_((data_*i*_-fit_*i*_)/*ε*_*i*_)^2^(*N*_idp_ − *N*_var_)^−1^). *N*_idp_, *N*_pts_ and *N*_var_ are, respectively, the number of independent points in the model fit (21), the total number of data points (201), and the number of fit variables (4–5). *ε*_*i*_ is the uncertainty of the *i*th data point.^68^

c Theoretical contributions calculated based on (co-)precipitate additions listed in on Table S2 for the treatments ^57^Fh, ^57^Fh^13^GluC, and ^57^Fe + GluC_aq_.

d Fe in clays = fit as 1Mt-1 (which contains ∼20% Fe(ii)) or for the ^57^Fe + GluC_aq_ treatment, fit as Fe(ii) in clay using a chemically reduced smectite SWa-1 (called here SWa-1_red) as a reference. Abbreviations: Fh = ferrihydrite. Gt = goethite. Lp = lepidocrocite. Fe(ii)-organic = fit as Fe(ii)-gluconate. Fe(iii)-organic = fit as Fe(iii)-citrate or Fe(iii)-oxalate.

Changes in solid-phase Fe oxidation state and speciation agree with the increases in adsorbed Fe(ii) (determined as Fe(ii) in the 0.5 M HCl extractions, Table S5†). For all treatments, adsorbed Fe(ii) increased over the incubation and was highest in the ^13^GluC_aq_ and ^57^Fh + GluC_aq_ treatments after 6 weeks (∼23 mg g^−1^), in agreement with LCF analysis of XANES spectra. Lower amounts of adsorbed Fe(ii) were determined in the ^57^Fh^13^GluC (18.9 ± 1.2 mg g^−1^), control (15.0 ± 0.8 mg g^−1^), and ^57^Fh (12.6 ± 0.5 mg g^−1^) treatments. In general, these trends agree with aqueous geochemical data (Fig. S3†) and indicate that the glucuronic acid added in the ^13^GluC_aq_ and ^57^Fh + GluC_aq_ treatments stimulated microbial activity, resulting in enhanced Fe(iii) reduction. This is consistent with the previously demonstrated rapid microbial utilization of the added ^13^GluC_aq_ in these treatments, while the coprecipitated glucuronic acid showed reduced bioavailability (Table 1),^17^ thus limiting Fe(iii) reduction in the ^57^Fh^13^GluC treatment. For the ^57^Fe + GluC_aq_ treatment, the high accumulation of adsorbed Fe(ii) and trends in aqueous geochemistry similar to the ^13^GluC_aq_ and ^57^Fh + GluC_aq_ treatments suggest that the microbial utilization of the glucuronic acid was likely rapid in this treatment as well.

### Re-distribution of the ^57^Fe atoms

**Fig. 1:**
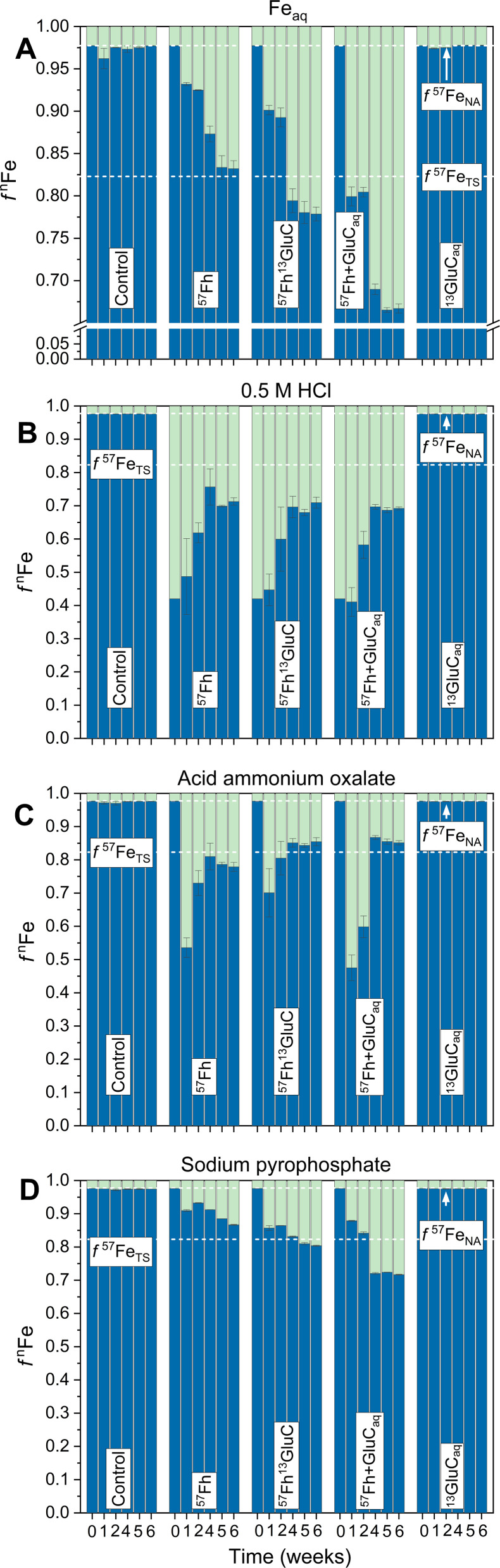
Trends in Fe isotope composition of Fe_aq_ (A) and the selective chemical extractions, shown as *f*^*n*^Fe, where *n* = 56 (blue) or 57 (green). (B) and (C) are steps 1 and 2 of the two-step sequential extraction whereby (B) is total Fe extracted with 0.5 M HCl (2 h) and (C) is Fe mobilized with an acid ammonium oxalate extraction. Panel (D) shows the isotope composition of Fe mobilized in the Na-pyrophosphate treatment. The initial timepoint (week 0) is calculated based on the experimental set-up (Table S2†). Dashed lines indicate the calculated isotope composition of the total system (TS) at equilibrium and initially (IN) directly following the addition of the isotope-labelled (co-)precipitates, and the natural abundance (NA) isotope composition considering the iron isotopes ^56^Fe and ^57^Fe only.^69^ Error bars indicate the standard deviation calculated from triplicate incubation bottles (A–C) or duplicate measurements of homogenized samples from triplicate incubation bottles (D). Panel A has, in part, been published in ref. 17.

The reductive dissolution and fate of ^57^Fe-labelled ferrihydrite in the ^57^Fh, ^57^Fh^13^GluC and ^57^Fh + GluC_aq_ treatments was tracked, in part, through its release to the aqueous phase, which resulted in changes in the iron isotope composition of Fe_aq_ (Fig. 1). Based on previous anoxic incubations of iron-rich organic Icelandic soils, Fe_aq_ is assumed to comprise primarily Fe(ii).^20^ In the absence of a ^57^Fe addition (Control and ^13^GluC_aq_ treatments), the fraction of ^57^Fe in Fe_aq_ (*f*^57^Fe_aq_) remained at the expected value for natural abundance iron isotope composition (∼2.3% ^57^Fe considering ^56^Fe and ^57^Fe only^69^). In contrast, in the ^57^Fh, ^57^Fh^13^GluC, and ^57^Fh + GluC_aq_ treatments, *f*^57^Fe_aq_ increased rapidly, reaching 6.8, 9.9, and 20.1% ^57^Fe (respectively) within 1 week and then continued to increase through week 4 of the incubation, indicating the reductive dissolution of the added ^57^Fe-labelled ferrihydrite in all treatments. Between weeks 5 and 6, *f*^57^Fe_aq_ plateaued at ∼16, 22, and 33% ^57^Fe for the ^57^Fh, ^57^Fh^13^GluC, and ^57^Fh + GluC_aq_ treatments, respectively. While *f*^57^Fe_aq_ in the ^57^Fh treatment was very near the iron isotope composition calculated for the total system (∼16.9% ^57^Fe considering ^56^Fe and ^57^Fe only,^69^ Table S2†), in the ^57^Fh^13^GluC and ^57^Fh + GluC_aq_ treatments it surpassed this value, indicating an enrichment of Fe_aq_ in ^57^Fe atoms in the presence of or in association with glucuronic acid. This may be explained by the formation of dissolved Fe(ii/iii)-organic complexes^70,71^ and/or the presence of ^57^Fe atoms in a fine colloidal fraction,^20^ both scenarios which may limit the re-adsorption of ^57^Fe atoms onto the soil matrix. In contrast, ^57^Fe released in the absence of glucuronic acid (^57^Fh treatment) re-sorbed to or was incorporated into soil constituents. However, it should be noted that the overall extent of Fe(iii) reduction was lowest in the ^57^Fh treatment (Table S5†) and thus additional ^57^Fe atoms might accumulate in the aqueous phase under prolonged reducing conditions. Still, the plateau in changes of Fe_aq_ iron isotope composition between weeks 5 and 6 suggests that accumulation of ^57^Fe atoms in solution was not limited by the experiment duration.

**Fig. 2:**
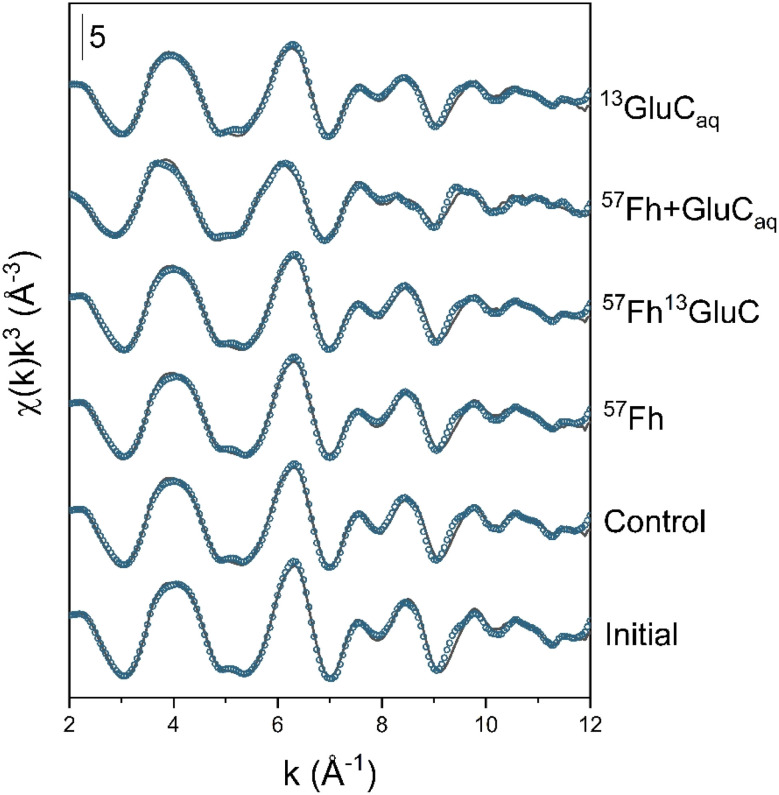
Iron K-edge EXAFS spectra of initial and incubated samples and their linear combination fits (LCF). Experimental data is shown as solid lines and model fits are shown as symbols. Fit results are reported in Table 2, reference spectra are shown in Fig. S5.†

Following changes in the iron isotope composition of selective chemical extractions revealed the time-resolved transformation of the ^57^Fe-labelled ferrihydrite (Fig. 1). Both the initial ^57^Fh and the ^57^Fh^13^GluC coprecipitate completely dissolved in the 0.5 M HCl extraction. Thus, that ^57^Fe atoms above NA contributions were observed in Fe_o_, the second extraction step, demonstrates that the added ^57^Fe-labelled ferrihydrite underwent transformation and/or recrystallization, forming a phase that was not extractable by 0.5 M HCl. Transformation of pure ferrihydrite to more thermodynamically stable mineral phases like goethite and magnetite is well documented in model studies (*e.g.*, ref. 24, 25 and 27–30) and has been similarly demonstrated during the Fe(ii)-catalyzed transformation of ferrihydrite–organic matter coprecipitates as well, albeit to a lesser extent with increasing C content.^1^ In agreement, the highest fraction of ^57^Fe atoms in Fe_o_ is seen in the ^57^Fh treatment (21% ^57^Fe at 6 weeks, corresponding to ∼32% of the added ^57^Fe-labelled ferrihydrite, Fig. 1) compared to the ^57^Fh + GluC_aq_ and ^57^Fh^13^GluC treatments (∼14% ^57^Fe at 6 weeks, corresponding to 21% and 12% of the added ^57^Fe-labelled ferrihydrite, respectively). In addition to transformation and/or recrystallization, the ^57^Fe-labelled ferrihydrite in all treatments may have been reductively dissolved, with a fraction of the ^57^Fe(ii) re-sorbing to the soil matrix (thus contributing to ^57^Fe in the 0.5 M HCl extraction or driving Fe(ii)-catalyzed recrystallization of soil minerals^58^) or contributing to the newly formed organically-complexed Fe(ii) (Table 2). The latter is strongly supported by the increase in ^57^Fe atoms mobilized in with Na-pyrophosphate (Fe_p_). Initially, neither the ^57^Fh nor the ^57^Fh^13^GluC coprecipitate were mobilized by Na-pyrophosphate and the steady increase in both total amounts of Fe (Table S5†) and the fraction of ^57^Fe (Fig. 1) in Fe_p_ are thus evidence of the transformation of the added ^57^Fe-labelled ferrihydrite.

### *In situ* transformation of (organic-associated) ^57^Fe-labelled ferrihydrite

In contrast to Fe K-edge XAS, in which the oxidation state and speciation of all Fe atoms in the sample are analyzed, ^57^Fe Mössbauer spectroscopy specifically targets ^57^Fe atoms. With the addition of ^57^Fe-labelled ferrihydrite to the soil in the ^57^Fh, ^57^Fh + GluC_aq_, and ^57^Fh^13^GluC treatments, ∼90% of the recorded Mössbauer signal could be attributed to iron atoms coming from the added ferrihydrite. Mössbauer spectra of the initial soil and the (co-)precipitates (*e.g.* ferrihydrite in ^57^Fh and the ferrihydrite–glucuronic acid coprecipitate ^57^Fh^13^GluC), measured at 77 and 5 K, and the initial mineral-soil mixtures (soil + ^57^Fh and soil + ^57^Fh^13^GluC), measured at 77, 45, 35, 25, 15, and 5 K, are shown in Fig. S7–S9.† The fit parameters are presented in the ESI (Tables S6 and S7, respectively)† with a detailed discussion on the fitted spectra that is summarized here. Briefly, Mössbauer spectra of the soil horizon used in the incubation study which, in the absence of added ^57^Fe-labelled ferrihydrite, is essentially a bulk analysis, agreed reasonably well with LCF analysis of Fe K-edge XANES and EXAFS spectra (compare Tables 2 and S7 to S3†). At 77 K, fits of Mössbauer spectra of the initial soil indicated 83% Fe(iii) and 17% Fe(ii), while 5 K Mössbauer spectra were fit with sextets and doublets indicating contributions from a mixture of ferrihydrite and goethite (27%), ferrihydrite precipitated in the presence of organic matter (32%), organically-complexed Fe(iii) or Fe(iii) in clays (13%), sorbed Fe(ii) or Fe(ii) in clays (5%), and a difficult-to-distinguish collapsed feature (23%), which may comprise low-crystallinity Fe oxides associated with organic matter, Al or Si^32,72^ and some Fe(ii).

Mössbauer spectra of the ^57^Fe-labelled ferrihydrite and the coprecipitate (^57^Fh and ^57^Fh^13^GluC) were similar at 77 K and 5 K (Fig. S8 and Table S7†). At 5 K, a slightly lower hyperfine field was required to fit the ferrihydrite in ^57^Fh^13^GluC (47.8 mm s^−1^*vs.* 48.5 mm s^−1^), in agreement with reports of ferrihydrite precipitated in the presence of organic matter.^31,37,73^ In agreement, a qualitative assessment of the magnitude of the Fourier transform *k*^3^-weighted Fe K-edge EXAFS spectra of the initial sample materials (Fig. S2†) revealed reduced amplitudes for ^57^Fh^13^GluC in features corresponding to corner- and edge-sharing Fe, similarly suggesting that coprecipitation with glucuronic acid impeded ferrihydrite crystal growth.^1^ Further evidence of structural differences between the ^57^Fe-labelled ferrihydrite and ferrihydrite in the ^57^Fe-labelled coprecipitate are seen in the temperature resolved Mössbauer spectra of the initial mineral-soil mixtures (*e.g.* soil + ^57^Fh and soil + ^57^Fh^13^GluC, Fig. S9†), which indicate that ferrihydrite in ^57^Fh was more magnetically ordered than ferrihydrite in the ^57^Fh^13^GluC (at 45 and 35 K), and thus is of higher crystallinity. It should be noted that Mössbauer spectra of the initial mineral-soil mixture in the ^57^Fh + GluC_aq_ treatment (*e.g.* soil + ^57^Fh + GluC_aq_) is assumed to be identical to that of the ^57^Fh treatment as the added ^57^Fe-labelled ferrihydrite was identical.

**Fig. 3:**
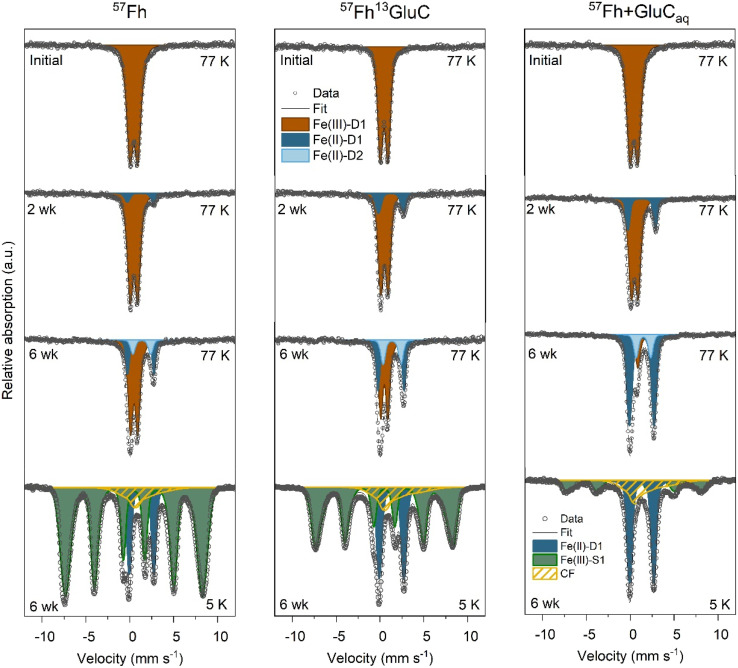
77 K Mössbauer spectra of ^57^Fh, ^57^Fh + GluC_aq_, and ^57^Fh^13^GluC treatments prior to incubation (top row), after 2 weeks incubation (upper middle row), after 6 weeks incubation (lower middle row) and 5 K Mössbauer spectra of 6 weeks incubated samples (bottom row). Complete temperature profile spectra are shown in Fig. S10† and fit parameters are detailed in Table S9.†

Solid-phase samples from the ^57^Fh, ^57^Fh^13^GluC and ^57^Fh + GluC_aq_ treatments after 2- and 6- weeks of anoxic incubation measured at 77 K show increasing fractions of solid-associated Fe(ii) over time (Fig. 3). At 2 weeks, a paramagnetic Fe(ii) doublet (Fe(ii)-D1; CS = ∼1.2 mm s^−1^ and quadrupole splitting QS = ∼2.9 mm s^−1^) was visible, accounting for 8%, 18%, and 21% of ^57^Fe atoms in the ^57^Fh, ^57^Fh^13^GluC, and ^57^Fh + GluC_aq_ treatments, respectively. At 6 weeks, the fraction of Fe(ii)-D1 increased to 21%, 31%, and 65% and a second paramagnetic doublet (Fe(ii)-D2; CS = ∼1.2 mm s^−1^ and QS = ∼2.9 mm s^−1^) was fit, accounting for an additional 10%, 17%, 15% of ^57^Fe atoms for the ^57^Fh, ^57^Fh^13^GluC, and ^57^Fh + GluC_aq_ treatments, respectively. Taken together, the fraction of ^57^Fe atoms attributed to Fe(ii) after 6 weeks according to Mössbauer spectroscopy was 31%, 48%, and 80% for the ^57^Fh, ^57^Fh^13^GluC, and ^57^Fh + GluC_aq_ treatments, respectively. In comparison, assuming that all of the 0.5 M HCl extractable Fe(ii) comprised ^57^Fe, the maximum fraction of ^57^Fe atoms attributed to Fe(ii) in the 0.5 M HCl extraction would be ∼27%, ∼41%, and ∼54% (Tables S4 and S5†). That higher amounts of ^57^Fe atoms in Fe(ii) were detected with Mössbauer than with the 0.5 M HCl extraction suggests that some of the Mössbauer-identified Fe(ii) was not extracted with 0.5 M HCl and thus may be in a more crystalline form, potentially contributing to the fraction of ^57^Fe atoms found in the Fe_o_ extraction step (Table S6†).

Identifying the nature of the ^57^Fe-phase found in the Fe_o_ extraction is difficult. Therefore, to aid in Fe species identification, solid-phase samples taken after 6 weeks of anoxic incubation were additionally measured at 45, 35, 25, 15, and 5 K. The temperature profile of spectra of these samples is shown in Fig. S10† and reveals that even at 5 K, the Fe(ii)-D1 phase is present as a paramagnetic doublet (15%, 25%, and 40%, for the ^57^Fh, ^57^Fh^13^GluC, and ^57^Fh + GluC_aq_ treatments, respectively), thus excluding its interpretation as siderite, a ferrous oxide, vivianite, or green rust (Néel temperatures of ∼37 K,^74^ ∼34 K,^75^ ∼12 K,^76^ and ∼5.2 K,^77^ respectively). Therefore, the Fe(ii)-D1 phase is likely Fe(ii) sorbed onto organic or mineral surfaces or Fe(ii) in clays.^78^ Spectra collected at the lower temperatures also revealed that part of the Fe(ii)-D2 component ordered between 35 and 77 K, (seen in the overall decrease in the total Fe(ii) observed as doublets at lower temperatures). Siderite orders into an octet at ∼37 K,^74^ and for both the ^57^Fh and ^57^Fh^13^GluC treatment samples, the fitting parameters of this Fe(ii)-D2 (CS = 1.36 mm s^−1^ and QS = 2.04 mm s^−1^) are consistent with siderite.^74^ However, the absence of a well-defined octet at 4.2 K fitted with parameters matching siderite allows us only to suggest that it may be a siderite-like phase. For the ^57^Fh + GluC_aq_ treatment sample, although the loss in area in the Fe(ii) doublet matches the Néel temperature of siderite, the fit parameters are not consistent with siderite. Spectra of the 6 weeks incubated samples also contained a broad sextet (Fe(iii)-S1) fit with parameters similar to those of the initial (co-)precipitate and the ferrihydrite fraction in the soil + ^57^Fh or soil + ^57^Fh^13^GluC spectra (CS = 0.47 mm s^−1^ and quadrupole shift *ε* = −0.01 mm s^−1^). However, the contribution of the Fe(iii)-S1 (ferrihydrite) phase decreased from ≥97% in the initial mineral-soil mixtures to 78%, 62%, and 29% in the 6 weeks incubated ^57^Fh, ^57^Fh^13^GluC, and ^57^Fh + GluC_aq_ treatments, respectively. Additionally, while the Fe(iii)-S1 (ferrihydrite) sextet in the ^57^Fh and ^57^Fh^13^GluC treatments had a similar hyperfine field as fitted in the initial (co-)precipitates (48.5 mm s^−1^ and 47.8 mm s^−1^, respectively), the hyperfine field of the Fe(iii)-S1 (ferrihydrite) phase in the ^57^Fh + GluC_aq_ treatment decreased from 48.1 mm s^−1^ to 47.6 mm s^−1^, suggesting that the residual ferrihydrite in this treatment was more similar to ferrihydrite found in ferrihydrite–organic matter coprecipitates.^32,38,40,48^ In addition, a collapsed feature accounted for 8%, 13%, and 31% of solid-phase ^57^Fe atoms in the ^57^Fh, ^57^Fh^13^GluC, and ^57^Fh + GluC_aq_ treatments, respectively. Although it is difficult to definitively state the composition of the collapsed feature, it likely comprises Fe(iii)-(oxyhydr)oxides near ordering temperature, such as low-crystallinity Fe oxides associated with organic matter, Al or Si^32,72^ and may include a siderite-like phase ordered into an octet.^74^

### Form of organic matter association influences the extent of reductive dissolution and transformation of ferrihydrite *in situ*

Collectively, Mössbauer results for the 6 weeks incubated samples showed that ^57^Fe-labelled ferrihydrite in the ^57^Fh^13^GluC treatment was both more reduced (48 *versus* 31% Fe(ii)) and transformed (62 *versus* 78% ferrihydrite remaining) compared to the pure ^57^Fh treatment. However, XAS results indicated minimal differences in bulk iron oxidation state and speciation between these samples; each containing similar amounts of Fe(ii) (13 and 16%), ferrihydrite (48 and 47%), goethite (18 and 17%), Fe in clays (27 and 26%), and organically-complexed Fe(iii) (8 and 10%), suggesting that, overall, Fe(iii) reduction proceeded similarly in both treatments. Thus, differences in the Mössbauer fitting results are attributed to varying characteristics and reactivity of the ferrihydrite in the ^57^Fh and ^57^Fh^13^GluC (co-)precipitates.

As indicated in the temperature-resolved Mössbauer spectra and XAS of the initial minerals and mineral-soil mixtures, ferrihydrite in the ^57^Fh was slightly more crystalline than ferrihydrite in the ^57^Fh^13^GluC coprecipitate. Nano-crystalline mineral phases like ferrihydrite tend to become thermodynamically more stable when particle size increases. Indeed, previous studies comparing the geochemical reactivity of pure ferrihydrite *versus* ferrihydrite–organic matter coprecipitates found increased susceptibility to ligand-promoted or abiotic reductive dissolution in the coprecipitates.^79,80^ This is likely due to OC-induced changes in mineral characteristics (*e.g.*, particle size, morphology, PZC)^1,31–35,37^ and structure.^1,33–35^ Glucuronic acid, a derivative of glucose, does not contain quinone moieties which could be used by microorganisms to shuttle electrons.^12,42–44^ However, a fraction of the glucuronic acid in the coprecipitate was easily desorbed (∼22% after 4 h in UPW), and measurements of the isotopic composition of CO_2_ produced in this system indicated this fraction to be rapidly utilized by the soil microorganisms.^17^ Moreover, mineralization of the native SOM in the ^57^Fh^13^GluC treatment was enhanced (priming effect) compared to the ^57^Fh treatment (or the control, Table 1).^17^ Thus, we suggest that the increased reductive dissolution of the ^57^Fe-labelled ferrihydrite in ^57^Fh^13^GluC treatment was likely due to both increased structural disorder as well as an active microbial community stimulated by the presence of glucuronic acid, some of which may have been easily accessed.

Yet, fitting from Mössbauer spectra also revealed that the ^57^Fe-labelled ferrihydrite in the ^57^Fh + GluC_aq_ treatment was both more reduced (80% Fe(ii)) and more transformed (29% ferrihydrite remaining) than the ^57^Fe-labelled ferrihydrite in either the ^57^Fh or ^57^Fh^13^GluC treatments. This agrees with bulk Fe XAS analysis, which indicated the largest changes in solid-phase Fe oxidation state in the ^57^Fh + GluC_aq_ treatment (Table S3†), and aqueous geochemical data, which recorded higher pH and lower Eh_7_ values in this treatment (Table 1 and Fig. S3†). These results indicate that the simultaneous but separate addition of the ^57^Fe-labelled ferrihydrite and glucuronic acid in the ^57^Fh + GluC_aq_ treatment resulted in the stimulation of microbial community which, in turn, led to faster and more complete reductive dissolution of Fe(iii) in the system, including the ^57^Fe-labelled ferrihydrite.

However, most interesting is that analysis of bulk Fe XAS spectra revealed that the extent of overall Fe(iii) reduction in the ^57^Fh + GluC_aq_ treatment surpassed the extent of Fe(iii) reduction recorded in any of the other treatments, including the ^13^GluC_aq_ treatment (Table 2 and S5†). In the ^13^GluC_aq_ treatment, soil microorganisms were supplied with an easily accessible electron donor, yet relied on existing electron acceptors in the soil matrix. In the ^57^Fh + GluC_aq_ treatment, soil microorganisms were supplied with both an easily accessible electron donor as well as an easily accessible electron acceptor. The high extent of reduction of the ^57^Fe-labelled ferrihydrite in addition to the enhanced reduction of native soil Fe recorded here suggests that the added ferrihydrite was a more accessible electron acceptor than the existing soil Fe phases, yet the combined addition of easily accessible electron acceptor and donor resulted in the highest stimulation of the microbial communities, thereby enhancing reduction of the native soil Fe(iii) as well.

### Soil matrix influences the products of ferrihydrite transformation *in situ*

The presence or absence of organic matter exerts control over the extent and products of ferrihydrite transformation in model studies. This has been demonstrated for OC-adsorbed and coprecipitated ferrihydrite in the presence of Fe(ii),^1,37,40,41^ S(-ii),^36,81^ and during dissimilatory iron reduction.^42,43,80^ Collectively, these studies suggest that, while transformation products of OC-free ferrihydrite tend to be crystalline (*e.g.* lepidocrocite, goethite, or magnetite), the presence of OC hinders ferrihydrite transformation. In this study, we found no evidence of typical crystalline ferrihydrite transformation products (*e.g.* lepidocrocite, goethite, or magnetite) in either the ^57^Fh or ^57^Fh + GluC_aq_ treatments, despite the ‘OC-free’ nature of the ^57^Fe-labelled ferrihydrite in these treatments. Instead, after 6 weeks of anoxic incubation, ^57^Fe atoms comprised similar phases in all treatments (Table S9†), none of which were distinctly crystalline (aside from small contributions from a siderite-like phase). This suggests that aqueous geochemical conditions and the soil matrix exerted more control over the transformation products than the presence or absence of coprecipitated glucuronic acid.

A lack of typical crystalline transformation products (*e.g.* lepidocrocite, goethite, magnetite), particularly in the ^57^Fh and ^57^Fh + GluC_aq_ treatments, may be explained by the high organic carbon content of the soil and the aqueous phase (Table S1 and Fig. S3†), or may also reflect the tendency of this soil to release ions to solution that are known to interfere with ferrihydrite transformation (*e.g.*, P,^82^ Al,^83^ Si;^84^ see ref. 20). The presence of various dissolved organic ligands, Si, or P may promote the formation of green-rust during microbial reduction of Fe(iii)-(oxyhydr)oxides,^82,85^ and geochemical modelling suggests that green-rust may be stable in Icelandic peat soils.^86^ Still, green-rust was not identified in this study. Instead, indications of siderite formation were seen in Mössbauer spectra of all treatments. While geochemical modelling suggests that siderite typically occurs at lower redox and higher pH conditions than those recorded in this study,^86^ small grains of siderite (10–30 μm) have previously been identified in thin sections from a soil profile located near the Hestur field site.^87^

## Environmental implications

In a previous study, coprecipitation of a LMWOa with ferrihydrite was found to limit bioavailability of the former in an anoxic soil.^17^ Here, we show that the limited bioavailability of the coprecipitated LMWOa likewise has implications for the reductive dissolution and transformation of the coprecipitated ferrihydrite, with cascading impacts on the reduction of native soil Fe(iii). Collectively, our results illustrate the importance of physical association of OC and minerals: despite the addition of identical amounts of electron acceptor (^57^Fe-labelled ferrihydrite) and electron donor ((^13^C-)glucuronic acid) in the ^57^Fh^13^GluC and ^57^Fh + GluC_aq_ treatments, the close physical association of the two in the coprecipitate did not lead to the enhanced utilization of Fe(iii) in the coprecipitated ferrihydrite, as may have been expected from model studies following microbial reductive dissolution of ferrihydrite-OC coprecipitates.^10,12,42–44^ Rather, coprecipitation limited the bioavailability of the mineral-associated glucuronic acid,^17^ which, in turn, limited the reductive dissolution and transformation of the coprecipitated ferrihydrite and had little impact on the reduction of the native soil Fe(iii). In contrast, the separate but simultaneous spike of the ^57^Fh + GluC_aq_ treatment resulted in overall enhanced microbial activity which led to not only higher extents of reductive dissolution of the ^57^Fe-labelled ferrihydrite, but also the reduction of significantly more native soil Fe(iii). For Icelandic wetland soils, the majority of which are minerotrophic^88^ and impacted by aeolian deposition of Fe-rich volcanic dust,^57^ our results imply that future spatial and temporal changes both groundwater flow and dust deposition in the proximity of these organic-rich soils will be relevant to understanding coupled biogeochemical cycling of carbon and iron in these soils. This may be especially in the context of climate change, which is driving ongoing glacial retreat, changing hydrologic regimes and leading to intensified aeolian processes.^89^

It could be noted that the C : Fe molar ratio of the coprecipitate in this study (0.42) is relatively low compared to C : Fe molar ratios found in natural ferrihydrite-rich-OC coprecipitates collected in Icelandic wetlands (0.4–2.4), which comprise more complex and heterogeneous organic functional groups.^53^ Changing both of these factors (increased C : Fe molar ratios and inclusion of complex, heterogeneous organic matter) may alter bioavailability of the coprecipitated OC and, in turn, impact the stability of the coprecipitated ferrihydrite. That the crystallinity and identity of the ferrihydrite transformation products were largely influenced by the soil matrix rather than the presence or absence of coprecipitated OC agrees with recent studies highlighting the importance of contact to the soil matrix.^54,90^ For this reason, it is important to note that the soil used in this study was high in both OC and iron mineral content. The impact of OC-coprecipitated mineral additions to soils may be different if the soil is OC or iron limited.

Finally, our results demonstrated that variations in individual mineral transformation pathways and extents is not necessarily reflected in bulk solid phase analysis techniques. However, the combination of bulk solid phase analyses (*e.g.* Fe K-edge XAS and selective chemical extractions) and the application of stable iron isotopes as tracers offers a promising approach to probe the fate of individual minerals within a soil matrix. Collectively, this research highlights the importance of a holistic understanding of the coupled biogeochemical cycles of iron and carbon in the context of natural soil.

## Data availability

The datasets generated and/or analyzed during the current study are available from the corresponding author on reasonable request. Other data are included in the ESI.†

## Conflicts of interest

There are no conflicts to declare.

## Supplementary Material

EM-026-D4EM00238E-s001
