# Supplementary material for: Emerging investigator series: Coprecipitation with glucuronic acid limits reductive dissolution and transformation of ferrihydrite in an anoxic soil
Source: Environ Sci Process Impacts. 2024 Jul 25;26(9):1489–502. doi: 10.1039/d4em00238e (PMC11409838; doi:10.1039/d4em00238e)
Supplement: EM-026-D4EM00238E-s001 [file EM-026-D4EM00238E-s001.pdf]

Electronic Supplementary Material (ESI) for Environmental Science: Processes & Impacts.
This journal is © The Royal Society of Chemistry 2024

Supporting Information to:

'Coprecipitation with glucuronic acid limits reductive dissolution and transformation of ferrihydrite in an anoxic soil'

Laurel K. ThomasArrigo,*[a,b] Luiza Notini,[b] Sophie Vontobel,[b] Sylvain Bouchet,[b] Tabea Nydegger,[b] Ruben Kretzschmar[b]

[a]*Environmental Chemistry Group, Institute of Chemistry, University of Neuchâtel, Avenue de Bellevaux 51, CH-2000, Neuchâtel, Switzerland*

[b]*Soil Chemistry Group, Institute of Biogeochemistry and Pollutant Dynamics, Department of Environmental Systems Science, ETH Zurich, Universitätstrasse 16, CHN, CH-8092 Zurich, Switzerland*

(26 Pages, 10 Figures, 9 Tables)

Table of contents

1. Soil profile location, description, and mineralogy S2
2. Synthesis and characterization of the ^{57}Fe-labelled (co-)precipitates S3
3. Experimental conditions S5
4. Aqueous- and solid-phase sampling procedure S6
5. X-ray absorption spectroscopy S8
6. Selective chemical extractions S11
7. ^{57}Fe Mössbauer spectroscopy S14
8. References S25

*Corresponding author: laurel.thomas@unine.ch

1. Soil profile location, description, and mineralogy

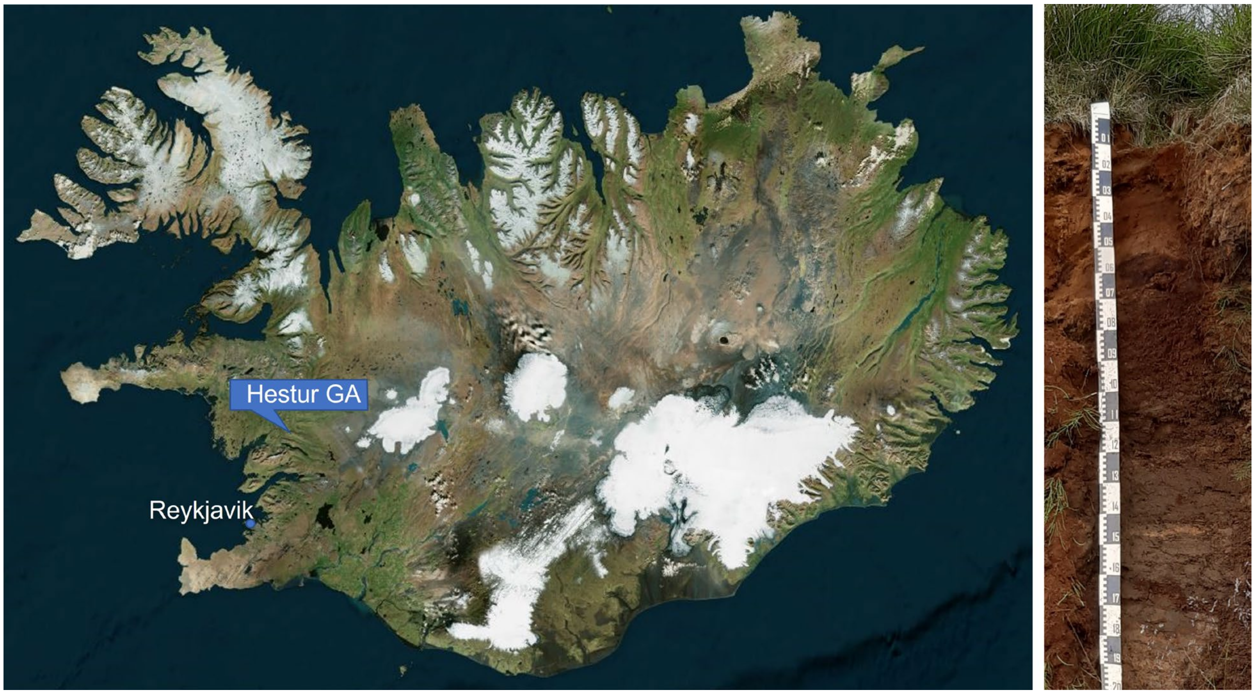

Figure S1. (L) Map of Iceland with soil profile sampling location. Orthoimage based on data from National Land Survey of Iceland. (R) The complete Hestur_GA (2020) soil profile.

Table S1. Physical and elemental characterization of the Hestur_GA (2020) soil profile.

Site Name	Depth[a] (cm)	Horizon	pH (H_20)[b]	C_T[c] (wt.%)	C/N[c] Mass ratio	T[d] Al (mg g^{-1})	T[d] Fe (mg g^{-1})	T[d] Si (mg g^{-1})
**Hestur_GA (2020)**	0-50	A		11.7	11.8	32.2	193.1	77.7
	50-60	O1		29.2	15.7	26.8	62.8	49.7
	**60-72**	**O2**	**4.56**	**21.6**	**15.9**	**30.7**	**73.1**	**66.0**
	72-80	B1		9.7	13.9	79.3	75.2	131.3
	80-100	O3		25.4	16.8	44.9	54.6	78.6
	100-120	O4		11.6	14.7	77.8	77.7	115.4
	120-180	B2		3.2	10.9	94.1	96.2	164.4
	180-200	B3		6.3	16.7	49.8	216.8	106.0
	>200	O5		32.5	16.6	24.8	69.3	38.0

[a]Below soil surface. [b]Measured in suspended soils (1:10 solid:solution ratio) after 1 h at room temperature. [c]Determined with an elemental analyzer (CNS). [d]Total element content (XRF). This data has been previously published in ref. 1.

Soils for characterization and the incubation study were collected in July 2020. The soil profile was described following FAO guidelines.[2] Individual horizons were manually homogenized and packaged in their field-moist state into plastic bags which were then stored at 4°C in the dark. Subsets of each soil horizon were air dried (30°C) and sieved (<2 mm, nylon) for characterization. Soil pH was determined after re-suspending the dried soil in UPW at a solid:solution ratio of 1:5 for 1 hr. Total element contents of each soil horizon were measured with energy-dispersive X-ray fluorescence (XRF) spectrometry (Spectro X-Lab 2000) and total C and N contents with an elemental analyzer (Vario MAX Cube, Elementar).

Mineral composition of the soil horizon was determined by powder X-ray diffraction (XRD, D8 Advance, Bruker). For these analysis, 30 °C dried and sieved soil was milled to ~50 µm using a disk swing mill. Milled soil material was analyzed as powder XRD in Bragg−Brentano geometry using Cu $K\alpha_{1,2}$ radiation (λ = 1.5418 Å, 40 kV, and 40 mA) and a high-resolution energy-dispersive 1-D detector (LYNXEYE). Diffractograms were recorded from 10° to 70°2θ with a step size of 0.02°2θ and 6 s acquisition time per step. The relative contributions of the crystalline mineral phases in the diffraction patterns were determined by Rietveld Quantitative Phase Analysis (QPA) using the TOPAS software (Version 5, Bruker AXS) in combination with published crystallographic structure files. Additionally, the amount of amorphous material was estimated by the internal standard method in the TOPAS software using aluminum oxide (Al_2O_3, Fluka) as the internal standard mixed into the soil at a mass ratio of 1:2 (Al_2O_3:soil).

2. Synthesis and characterization of the ^{57}Fe-labelled (co-)precipitates

A description of the synthesis of isotope-labelled ferrihydrite (^{57}Fh) and the ferrihydrite-glucuronic acid coprecipitate (^{57}Fh^{13}GluC) using ^{57}Fe-labelled Fe(0) metal powder and ^{13}C-labelled glucuronic acid and a detailed description of the resulting (co-)preciptiates has been previously published.[1] Briefly, an ^{57}Fe(III) stock solution was prepared by dissolving ^{57}Fe(0) metal powder (95.08% ^{57}Fe, Isoflex, USA) in 2 M HCl (Normatron®, VWR) followed by oxidation with H_2O_2. For the synthesis of ^{57}Fh, the ^{57}Fe(III) stock solution was titrated with 1 M NaOH (Titrisol®) under vigorous stirring (1200/min) until a pH of 7.1 ± 0.1 was reached. For the synthesis of ^{57}Fh^{13}GluC, ^{13}C-labelled glucuronic acid (^{13}GluC, 99.99% ^{13}C, D-[UL-$^{13}C_6$]glucuronic acid sodium salt monohydrate, Omicron Biochemicals) was equilibrated overnight in darkness in 1 L UPW water adjusted to pH 7.0 with 1 M NaOH under vigorous stirring (1200/min). The ^{13}C-glucuronic acid-containing solution was then acidified to pH 4.0 with 1 M HNO_3 (Normatron®, VWR) and purged with N_2(g) for 15 min. After adding an aliquot of the ^{57}Fe(III) stock solution, the solution was titrated with 1 M NaOH as described in the synthesis of ferrihydrite. The mineral suspensions were

then centrifuged at 3600 *g* for 15 minutes, decanted, and re-suspended in UPW three times until the conductivity of the supernatant was <350 µS/cm. Afterwards, the suspensions were shock frozen by dropwise injection into liquid N_2 and freeze dried, homogenized with a mortar and pestle, and stored in brown glass bottles in a desiccator until use. Detailed characterization of the resulting solid phases, including total element content, the fraction of easily-desorbed C in the coprecipitate, and confirmation of the mineral phases present using powder XRD, has been previously published.[1] Briefly, the C:Fe molar ratio of the ferrihydrite-glucuronic acid coprecipitate $^{57}Fh^{13}GluC$ was 0.42 and ~10 mg g^{-1} C was easily-desorbed, accounting for ~22% of total C in the coprecipitate. For both ^{57}Fh and $^{57}Fh^{13}GluC$, XRD patterns confirmed the presence of 2-line ferrihydrite, visible as broad maxima around 2.54 and 1.49 Å.

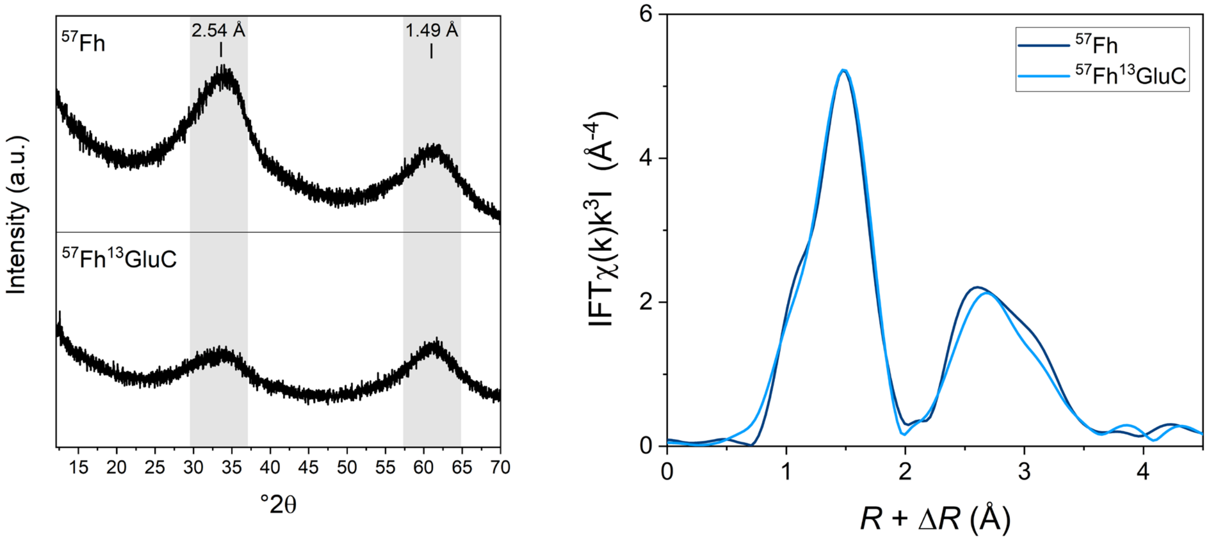

Figure S2. (Left) X-ray diffraction patterns of ^{57}Fh and $^{57}Fh^{13}GluC$. (Co)precipitates show the broad peak features at 2.54 and 1.49 Å typical of 2-line ferrihydrite. Figure published in ref. [1]. (Right) Magnitudes of the Fourier transform k^3-weighted Fe *K*-edge EXAFS spectra of ^{57}Fh and $^{57}Fh^{13}GluC$. Qualitative comparison suggests that the coprecipitate has lower amplitudes for features corresponding to corner- and edge-sharing Fe.

3. Experimental conditions

Table S2. Experimental conditions.[a]

Treatment	Dry soil weight	Total[b] native Fe	Total[c] native ^{57}Fe	Total[d] native C	Total[e] native ^{13}C	Spike[f]	^{57}Fe added	Increase in total soil Fe content	Theoretical total system $^{56/57}$Fe ratio	Mössbauer signal from added ^{57}Fe	$^{(13-)}$C added	Increase in total soil C content	Theoretical change in total system δ^{13}C
	(g per bottle)	(mg per bottle)						(%)	(-)	(%)	(mg)	(%)	(‰)
**Control**	3.5	256	5.42	756	8.09	0	0	0	43.28	0	0	0	0
**^{57}Fh**	3.5	256	5.42	756	8.09	81.08	45	16.1	4.87	89.3	0	0	0
**^{57}Fh^{13}GluC**	3.5	256	5.42	756	8.09	88.06	45	16.1	4.87	89.3	4.09	0.45	+506.1
**^{57}Fh+GluC$_{aq}$**	3.5	256	5.42	756	8.09	81.08 / 11.04	45	16.1	4.87	89.3	4.09	0.45	0
**^{13}GluC$_{aq}$**	3.5	256	5.42	756	8.09	13.70	0	0	43.28	0	4.09	0.45	+506.1

[a]Soil:water ratio 1:10. Experiments were conducted in triplicate. [b]Based on XRF. [c]Based on natural Fe isotope abundance (f^{57}Fe = 2.12%).[3] [d]Based on an elemental analyzer (CNS). [d]Based on natural C isotope abundance (f^{13}C = 1.07%).[4] [f]Either ^{57}Fh, ^{57}Fh^{13}GluC, or $^{(13-)}$GluC.

4. Aqueous- and solid-phase sampling procedure

To prevent accumulation of CO_2 in the headspace of the septum bottles between samplings, the headspace was purged with humidified N_2 gas at a flow rate of 750 mL min^{-1} for 10 minutes every 2-4 days during the entire experiment. During the purging, the bottles were placed on an orbital shaker (150 rpm) at room temperature. After 72 h and 1, 2, 4, 5, and 6 weeks, following the purging of the headspace, the septum bottles were moved into the glovebox, where they were opened for anoxic sampling. First, pH and Eh (reported as Eh_7; the redox potential converted to pH 7) were measured directly in the soil slurry. Then, the bottles were then manually agitated to ensure resuspension of all soil particles and ~5 mL of the soil slurry was poured into 15 mL Falcon tubes which were then capped, wrapped in Parafilm, and removed from the glovebox for centrifugation (3000 *g* for 15 minutes). The centrifuged tubes were returned to the glovebox, the supernatant pipetted off and additionally filtered (<0.45 µm, nylon) and acidified for further aqueous analyses (described below). To ensure the removal of all aqueous Fe(II), the residual solid-phase was then resuspended by adding 5 mL of anoxic UPW to the Falcon tube and manually shaking it. The Falcon tubes were then again capped, wrapped in Parafilm, and removed from the glovebox for centrifugation (3000 *g*, 15 minutes), then returned to the glovebox. The supernatant was pipetted off and the residual solid phase allowed to dry in the glovebox atmosphere in the dark (<24 h). Solid-phase samples were then manually homogenized with a mortar and pestle and stored in the dark in the glovebox until further analyses. After sampling, the septum bottles were then re-capped and removed from the glovebox and returned to the orbital shaker (150 rpm) at 25°C.

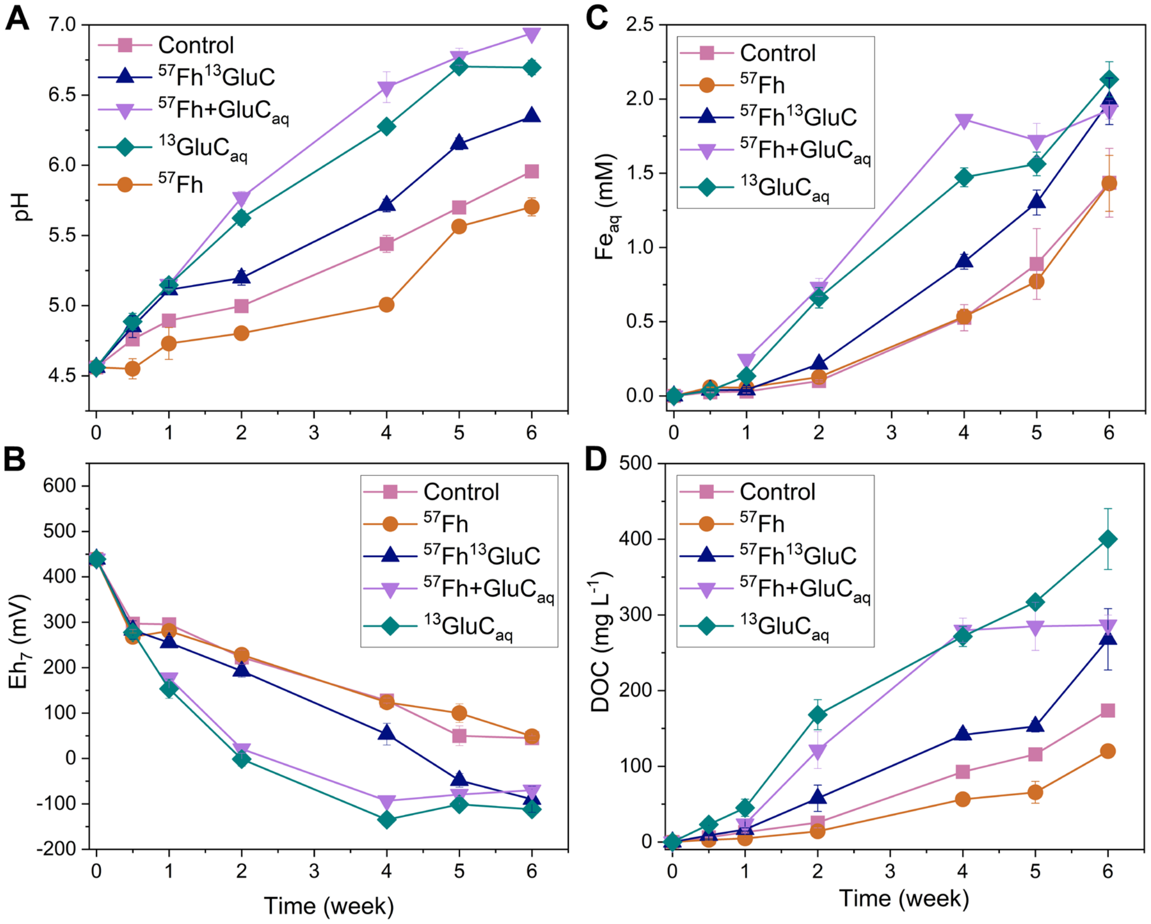

Figure S3. Aqueous geochemical data. Trends in pH (A), redox potential (Eh_7; Eh calculated relative to pH 7) (B), aqueous Fe (Fe_{aq}; panel C), and dissolved organic carbon (DOC; panel D) concentrations. Error bars indicate the standard deviation calculated from triplicate incubation bottles. Parts of this data have been previously published in ref. 1.

5. X-ray absorption spectroscopy

Iron *K*-edge (7112 eV) X-ray absorption spectroscopy (XAS) spectra were collected at the XAFS beamline of ELETTRA (Trieste, Italy) and at BM23 of ESRF (Grenoble, Italy). At ELETTRA, X-ray absorption near edge structure (XANES) and extended X-ray absorption fine structure (EXAFS) spectra were recorded in transmission mode at ~80 K using a N_2(l) cryostat. Higher harmonics in the beam were eliminated by detuning the monochromator by 30% of its maximal intensity and two to four scans were collected and averaged. At ESRF, spectra were recorded in transmission mode at ~10 K using a He(l) cryostat and higher harmonics in the beam were eliminated by mirrors. At both beamlines, the Si(111) monochromator was calibrated to the first-derivative maximum of the *K*-edge absorption spectrum of a metallic Fe foil (7112 eV). The foil was continuously monitored to account for small energy shifts (<1 eV) during the sample measurements.

All spectra were energy calibrated, pre-edge subtracted, and post-edge normalized in Athena.[5] Linear combination fit (LCF) analyses of Fe *K*-edge XANES spectra were conducted over an energy range of -20 to 30 eV (E-E_0) with E_0 of sample and reference compound spectra defined as the highest peak in the first XANES derivatives. Linear combination fit analyses of k^3-weighted Fe *K*-edge EXAFS spectra were performed over a *k*-range of 2-12 Å^{-1} with the E_0 of all spectra and reference compounds set to 7128 eV. No constraints were imposed during LCF analyses, and initial fit fractions (XANES: 101±1%, EXAFS: 88±5%) were recalculated to a compound sum of 100%.

Iron-containing reference compounds used for LCF analysis were selected based on previous measurements of similar soils from Iceland.[6] Visual comparison of the E_0 of the illite (1M1-1) reference sample suggested the sample contains both Fe(II) and Fe(III). This was confirmed with LCF, which showed that 1Mt-1 contained ~20% Fe(II) (Figure S6).

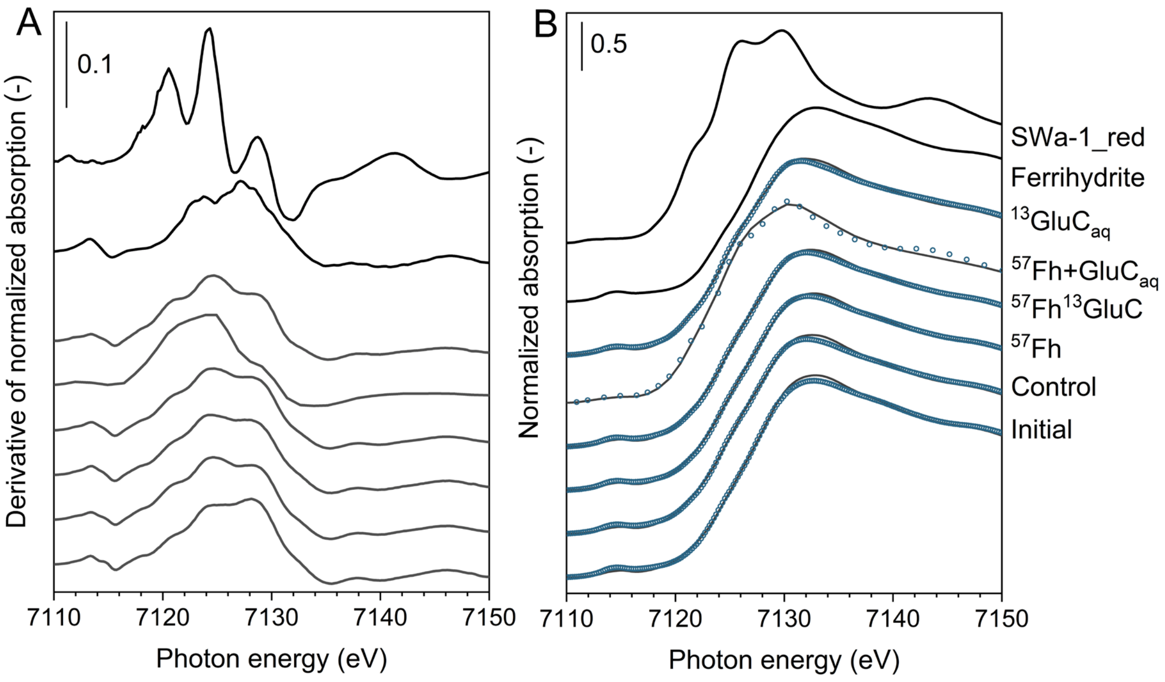

Figure S4. (A) First derivatives of normalized Fe *K*-edge XANES spectra of reference spectra and incubated soil samples and (B) LCF fits of the normalized spectra. Experimental data and model fits are shown as solid lines and symbols, respectively. Fit results are reported in Table S3.

Table S3. Linear combination fit results for Fe *K*-edge XANES spectra after 6 weeks anoxic incubation.[a]

Sample	Fe(III)	Fe(II)	NSSR[b]	red. χ^{2c}
	(%)			
**Initial**	94	6	0.15	0.0004
**Initial soil + ^{57}Fh**[d]	95	5		
**Control**	84	16	0.09	0.0002
**^{57}Fh**	87	13	0.05	0.0001
**^{57}Fh^{13}GluC**	84	16	0.06	0.0002
**^{57}Fh+GluC$_{aq}$**	44	56	0.18	0.0005
**^{13}GluC$_{aq}$**	75	25	0.08	0.0002

[a]Fe(III) and Fe(II) were fit by the references ferrihydrite and SWA-1_red, respectively. [b]Normalized sum of squared residuals ($100\sum_i(\text{data}_i-\text{fit}_i)^2/\sum_i\text{data}^2$). [c]Fit accuracy; reduced $\chi^2 = (N_{idp}/N_{pts})\sum_i((\text{data}_i-\text{fit}_i)/\varepsilon_i)^2(N_{idp}-N_{var})^{-1}$. N_{idp}, N_{pts} and N_{var} are, respectively, the number of independent points in the model fit, the total number of data points (249 or 38 for ^{57}Fh+GluC$_{aq}$), and the number of variables in the fit (2). ε_i is the uncertainty of the ith data point. Initial fit fractions (101±1%) were recalculated to 100%. [d]Theoretical contributions calculated based on (co-)precipitate additions listed in Table S2.

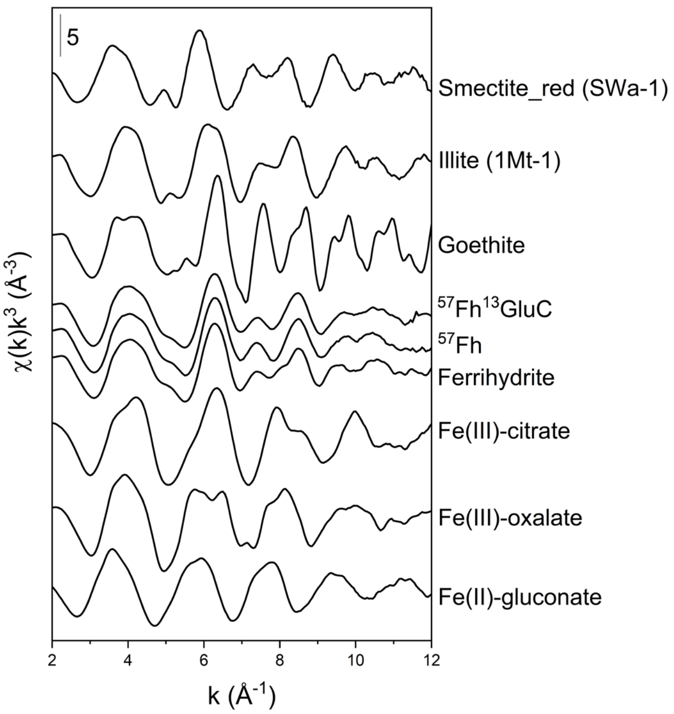

Figure S5. k^3-weighted Fe *K*-edge EXAFS spectra of references used in linear combination fits (LCF). ^{57}Fh and ^{57}Fh^{13}GluC are included here for comparison but were not used in LCF analyses.

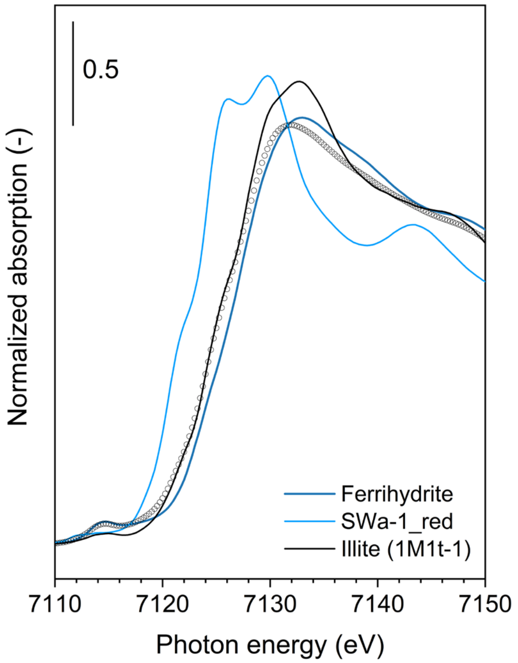

Figure S6. LCF of Fe *K*-edge XANES spectra of the illite (1Mt-1) reference used here. Results indicated that the reference contained ~20 % Fe(II). Experimental data and model fits are shown as solid lines and symbols, respectively.

Table S4. Fractions of Fe(II) fit with LCF analysis of XANES and EXAFS spectra.

Sample	XANES	EXAFS[a]
	(%)	
**Initial**	6	6
**Control**	16	6
**^{57}Fh**	13	5
**^{57}Fh^{13}GluC**	16	5
**^{57}Fh+GluC$_{aq}$**	56	50
**^{13}GluC$_{aq}$**	25	16

[a]Note that fractions of Fe(II) based on LCF of EXAFS spectra were determined assuming the Fe in clay fraction contained 20% Fe(II) and 80% Fe(III). See Figure S6 and discussions above.

6. Selective chemical extractions

The addition of ^{57}Fe-labelled ferrihydrite in the ^{57}Fh, ^{57}Fh^{13}GluC, and ^{57}Fh+GluC$_{aq}$ treatments led in higher initial amounts of 0.5 M HCl-extractable Fe, which stayed relatively stable for the duration of the incubation (Δ = ±20 % after 6 weeks). In contrast, amounts of 0.5 M HCl-extractable Fe in the Control and ^{13}GluC$_{aq}$ treatments steadily increased over the incubation (Δ = +47 and +97 % at 6 weeks, respectively). After 6 weeks, amounts of 0.5 M HCl-extractable Fe followed the order ^{57}Fe+GluC$_{aq}$ > ^{57}Fe^{13}GluC > ^{13}GluC$_{aq}$ > ^{57}Fh > Control.

The total amount of Fe in poorly-crystalline or amorphous mineral form (Fe$_o$) remained relatively stable over the incubation, with minor increases (Control, ^{57}Fh^{13}GluC and GluC$_{aq}$ treatments) and decreases (^{57}Fh, ^{57}Fh+GluC$_{aq}$ treatments) noted. The lack of clear trends agrees with previous anoxic incubations of a similar soil horizon (Hestur_GA_45-60 in ref. [6]), where only minor increases in Fe$_o$ were recorded (note that in ref. [6], extractions were not sequential). In contrast to Fe$_o$, trends in Fe$_p$; organically-bound or colloidal Fe, were more easily discernible and increased across all treatments during the incubation. For the ^{13}GluC$_{aq}$ and ^{57}Fh+GluC$_{aq}$ treatments, the increase in Fe$_p$ may be linked to the newly formed fraction of organically-complexed Fe(II) indicated by LCF analysis of EXAFS spectra (Table 1). It is possible that organically-complexed Fe(II) also formed in the other treatments, however contributions were less than our accepted detection limit for LCF analysis of EXAFS spectra (5 %). Increases in Fe$_p$ in all treatments may also be linked to colloidal Fe, as previous anoxic incubations recorded the formation of iron- and organic-rich fine colloids (3 kDa to 0.45 μm).[6]

Table S5. Results from selective chemical extractions.

Treatment	Time	0.5 M HCl extractable Fe(II)[a,b]	0.5 M HCl extractable Fe[b]	Fe_o[c]	Fe_p[d]
		mg g^{-1}			
**Initial soil**			11.8 (1.4)	23.0 (1.1)	20.8 (2.2)
**Initial soil + ^{57}Fh**[e]			~24.0[e]	-[e]	-[e]
**Control**	1 wk	2.9 (0.8)	10.1 (0.3)	13.8 (0.8)	25.8 (0.2)
	2 wk	3.6 (1.5)	11.6 (0.7)	13.0 (2.7)	27.6 (1.0)
	4 wk	7.7 (0.8)	14.0 (0.4)	16.5 (0.5)	31.2 (1.0)
	5 wk	10.9 (1.4)	16.4 (0.7)	19.1 (1.4)	28.6 (0.5)
	6 wk	15.0 (0.8)	17.4 (0.6)	20.2 (2.9)	30.8 (0.0)
**^{57}Fh**	1 wk	4.0 (0.5)	23.4 (7.0)	25.0 (1.6)	25.5 (0.5)
	2 wk	5.3 (0.7)	22.9 (3.8)	20.7 (5.1)	26.4 (0.4)
	4 wk	7.7 (0.6)	18.3 (1.9)	19.1 (2.2)	29.6 (0.3)
	5 wk	8.8 (0.5)	19.9 (0.2)	19.8 (2.1)	27.5 (1.0)
	6 wk	12.6 (0.5)	20.1 (0.8)	20.1 (2.3)	28.0 (0.3)
**^{57}Fh^{13}GluC**	1 wk	6.3 (0.3)	23.5 (5.6)	14.6 (4.6)	26.1 (0.1)
	2 wk	9.4 (1.9)	30.6 (2.8)	22.8 (2.3)	28.6 (0.4)
	4 wk	12.7 (1.1)	23.2 (2.2)	18.3 (2.9)	31.9 (0.3)
	5 wk	14.8 (0.3)	23.3 (1.0)	17.3 (0.7)	32.1 (1.2)
	6 wk	18.9 (1.2)	25.8 (0.4)	20.0 (0.3)	29.7 (1.1)
**^{57}Fh+GluC$_{aq}$**	1 wk	4.2 (0.2)	22.2 (1.3)	13.9 (3.7)	19.0 (0.1)
	2 wk	6.9 (0.9)	15.1 (0.9)	12.7 (0.5)	21.0 (0.5)
	4 wk	18.5 (2.4)	26.6 (2.3)	14.5 (0.9)	30.1 (0.1)
	5 wk	22.4 (1.1)	27.9 (0.6)	16.6 (1.1)	27.4 (4.5)
	6 wk	23.6 (1.3)	29.7 (1.9)	11.5 (0.8)	30.7 (0.9)
**^{13}GluC$_{aq}$**	1 wk	9.4 (6.7)	10.4 (0.8)	15.2 (1.0)	26.9 (0.0)
	2 wk	12.7 (0.2)	10.9 (1.0)	13.1 (1.4)	32.4 (0.4)
	4 wk	18.2 (1.3)	18.3 (0.9)	16.6 (1.1)	36.6 (0.5)
	5 wk	20.1 (1.9)	20.7 (1.8)	16.7 (3.3)	30.8 (2.0)
	6 wk	23.6 (1.4)	23.3 (1.0)	17.8 (3.1)	35.4 (1.3)

[a]As determined by the 1,10-phenanthroline method[7,8] in ([b]) 0.5 M HCl extractions[9] as the first step of a 2-step sequential extraction method. [c]Acid ammonium oxalate extraction[7] as the second step of a 2-step sequential extraction method. [d]A sodium-pyrophosphate treatment[10] was conducted on separate samples. [e]Theoretical contributions calculated for ^{57}Fh, ^{57}Fh^{13}GluC and ^{57}Fh+GluC treatments based on extractions of the (co-)precipitates, whereby Fe in the ^{57}Fh and the ^{57}Fh^{13}GluC coprecipitate was completely (>98%) mobilized in the 0.5 M HCl extraction and was hardly mobilized (<4%) by the sodium-pyrophosphate treatment. Errors in parenthesis represent the standard deviation of triplicate incubation bottles for the 0.5 M HCl and acid ammonium oxalate extractions. For the sodium-pyrophosphate treatment, solid phases from the triplicate incubation bottles were combined and the treatment was conducted in duplicate (error shown in parenthesis).

Table S6. Iron isotope mass balance.

Treatment	Time	Fe_{aq} (mg g^{-1} soil)			0.5 M HCl-extractable Fe (mg g^{-1} soil)			Fe_o (mg g^{-1} soil)			Fe_p (mg g^{-1} soil)			Recovery
		total	$f\,^{57}Fe^{a}$	% ^{57}Fe in Fe_{aq}[a]	total	$f\,^{57}Fe$	% ^{57}Fe in Fe_{HCl}	total	$f\,^{57}Fe$	% ^{57}Fe in Fe_o	total	$f\,^{57}Fe$	% ^{57}Fe in Fe_p	0.5 M HCl Fe + Fe_o
**Control**	1 wk	0.016	0.0379		10.1	0.0225		13.8	0.0269		25.8	0.0238		
	2 wk	0.057	0.0248		11.6	0.0226		13.0	0.0282		27.6	0.0265		
	4 wk	0.294	0.0267		14.0	0.0223		16.5	0.0233		31.2	0.0244		
	5 wk	0.497	0.0250		16.4	0.0223		19.1	0.0230		28.6	0.0242		
	6 wk	0.802	0.0227		17.4	0.0227		20.2	0.0229		30.8	0.0242		
**^{57}Fh**	1 wk	0.031	0.0680	0.015	23.4	0.4936	89.8	25.0	0.4459	86.7	25.5	0.0855	16.9	1.77
	2 wk	0.071	0.0750	0.038	22.9	0.3649	65.0	20.7	0.2512	40.5	26.4	0.0638	13.1	1.06
	4 wk	0.299	0.1268	0.257	18.3	0.2315	33.0	19.1	0.1681	25.0	29.6	0.0831	19.1	0.58
	5 wk	0.431	0.1663	0.464	19.9	0.2870	44.4	19.8	0.1923	29.7	27.5	0.1087	23.3	0.74
	6 wk	0.800	0.1678	0.869	20.1	0.2737	42.8	20.1	0.1973	30.8	28.0	0.1262	27.5	0.74
**^{57}Fh^{13}GluC**	1 wk	0.023	0.0987	0.016	23.5	0.5328	97.4	14.6	0.2886	32.8	26.1	0.1353	27.4	1.30
	2 wk	0.120	0.1073	0.089	30.6	0.3837	91.3	22.8	0.1805	32.1	28.6	0.1284	28.6	1.23
	4 wk	0.505	0.2057	0.641	23.2	0.2895	52.2	18.3	0.1329	18.9	31.9	0.1599	39.7	0.71
	5 wk	0.728	0.2196	0.970	23.3	0.3055	55.4	17.3	0.1406	18.9	32.1	0.1803	45.0	0.74
	6 wk	1.108	0.2213	1.486	25.8	0.2772	55.6	20.0	0.1296	20.1	29.7	0.1861	43.0	0.76
**^{57}Fh+GluC$_{aq}$**	1 wk	0.138	0.2007	0.214	22.2	0.5681	97.9	13.9	0.3264	30.2	19.0	0.1143	16.9	1.44
	2 wk	0.409	0.1955	0.621	15.1	0.3998	47.1	12.7	0.4715	40.0	21.0	0.1500	24.5	0.80
	4 wk	1.040	0.3102	2.509	26.6	0.2892	59.9	14.5	0.2869	29.3	30.1	0.2659	62.3	0.73
	5 wk	0.961	0.3347	2.502	27.9	0.2989	64.9	16.6	0.1273	15.1	27.4	0.2625	56.0	0.81
	6 wk	1.076	0.3329	2.785	29.7	0.2935	67.7	11.5	0.1382	11.5	30.7	0.2696	64.3	0.80
**^{13}GluC$_{aq}$**	1 wk	0.075	0.0258		10.4	0.0225		15.2	0.0232		26.9	0.0234		
	2 wk	0.369	0.0250		10.9	0.0226		13.1	0.0229		32.4	0.0236		
	4 wk	0.823	0.0223		18.3	0.0231		16.6	0.0227		36.6	0.0237		
	5 wk	0.873	0.0222		20.7	0.0225		16.7	0.0228		30.8	0.0234		
	6 wk	1.191	0.0222		23.3	0.0227		17.8	0.0230		35.4	0.0233		

[a] ^{57}Fe fractions in Fe_{aq} were determined in terms of ^{56}Fe to ^{57}Fe only and therefore are not included in the iron isotope recovery. All other ^{57}Fe fractions are reported relative to the iron isotopes ^{54}Fe, ^{56}Fe, ^{57}Fe and ^{58}Fe.

7. ^{57}Fe Mössbauer spectroscopy

7.1 Hestur_GA 60-72 soil horizon

The soil horizon selected for this incubation study (from the Hestur_GA (2020) soil profile, 60-72 cm depth) was analyzed with Mössbauer spectroscopy 77 K and 5 K (Figure S6, Table S7). The 77 K spectrum was composed of an Fe(II) doublet, an Fe(III) doublet, and an Fe(III) sextet which account for 17 %, 18 %, and 65 % of Fe atoms, respectively.

The fitting of the 5 K spectrum revealed two Fe(III) sextets (called Fe(III)-S1 and Fe(III)-S2), an Fe(II) doublet, an Fe(III) doublet, and a collapsed feature (CF). The first Fe(III) sextet, Fe(III)-S1, contributed to 27 % of the spectrum and had a CS = 0.49 mm s^{-1}, ε = -0.11 mm s^{-1}, and H = 49.4 T; parameters that could be considered intermediate between ferrihydrite and goethite[11] and therefore likely corresponds to a mixture of these two minerals. The second sextet, Fe(III)-S2, contributed to 32 % of the spectrum and had fitting parameters compatible with lepidocrocite: CS = 0.50 mm s^{-1}, ε = 0.00 mm s^{-1} and H = 45.3 T.[11] Alternatively, the high carbon content of this soil (21.6 wt.%) suggests that the Fe(III)-S2 sextet is ferrihydrite coprecipitated in the presence of dissolved organic matter, which has been shown to cause significant decreases in the hyperfine field.[12] The collapsed phase, CF, observed in the 5 K spectra, accounted for 23 % of the spectrum and contained Fe oxyhydroxides near their ordering temperatures, thus assigning them to individual mineral phases is not possible. However, similar Fe phases have been suggested to consist of organic matter-mineral associations[12] or Fe minerals associated with Al or Si.[13] The Fe(II) doublet, contributing to 5 % of the spectrum, had fitting parameters that could be compatible with Fe(II) in clays or Fe(II) sorbed onto Fe minerals: CS = 1.20 mm s^{-1} and QS = 2.27 mm s^{-1}.[14] However, the loss in the area of the Fe(II) doublet in the 5 K spectrum compared to the 77 K spectrum, combined with the high center shift value of the collapsed feature (0.73 mm s^{-1}) suggests the area assigned to the collapsed feature may additionally contain Fe(II) minerals that are ordered into an octet indistinguishable from the rest of the collapsed feature. The Fe(III) doublet, Fe(III)-D1, contributed to 13 % of the spectrum and had fitting parameters compatible with monomeric Fe(III)-OM or Fe(III) in clays: CS = 0.48 mm s^{-1} and QS = 0.75 mm s^{-1}.[15]

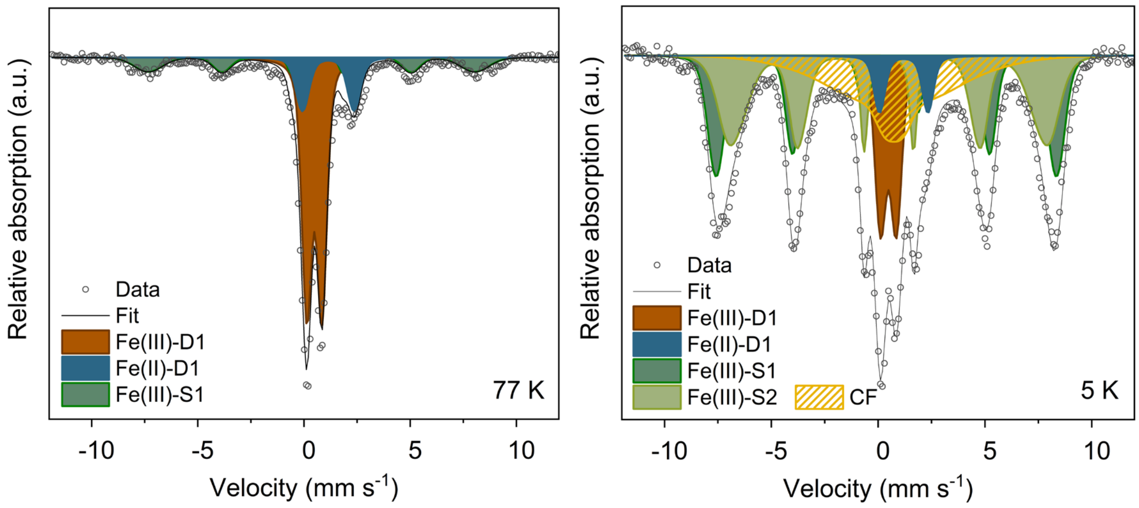

Figure S7. Mössbauer spectra of the soil used in the incubation (Hestur_GA 60-72, Table S1) collected at 77 K (left) and 5 K (right). Fit parameters are detailed in Table S7.

6.2 ^{57}Fh and ^{57}Fh^{13}GluC (co-)precipitates

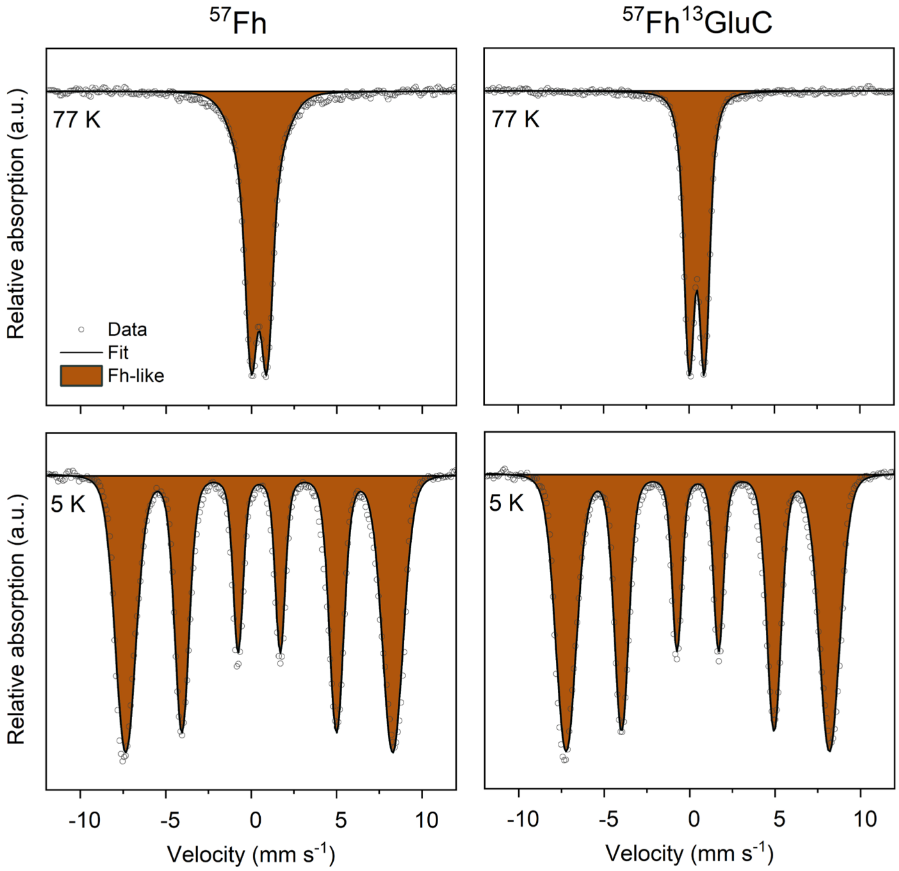

Figure S8. Mössbauer spectra of the (co-)precipitates, ^{57}Fh and ^{57}Fh^{13}GluC, collected at 77 and 5 K. Fit parameters are detailed in Table S7.

6.3 Initial soil + ^{57}Fh or ^{57}Fh^{13}GluC

Mössbauer spectra of the unreacted soil + ^{57}Fh or ^{57}Fh^{13}GluC at all temperatures were dominated by the added ferrihydrite and most the components attributed to the soil could no longer be distinguished (compare to the spectra of the Hestur_GA soil in Figure S6). At 77 K, the spectra were fit with an Fe(III) doublet (Fe(III)-D) of CS = 0.46 mm s^{-1} and QS = 1.06 mm s^{-1} (soil + ^{57}Fh) and CS = 0.46 mm s^{-1} and QS = 0.95 mm s^{-1} (soil + ^{57}Fh^{13}GluC). At 5 K, the spectra were also similarly composed of a broad Fe(III) sextet corresponding to Fh[16] and magnetically ordered components originating in the soil (≥97% of ^{57}Fe atoms) and a small Fe(III) doublet component most likely originating from the soil (see Table S8).

Considering that the soil matrix in both treatments is identical and only minorly contributes to the overall Mössbauer signal (~10 %), differences in fitted parameters are attributed to varying characteristics of the ^{57}Fe-labelled ferrihydrite in the ^{57}Fh and the ^{57}Fh^{13}GluC (co-)precipitates. For example, the fitted mean hyperfine field of the Fe(III)-S1 sextet matching ferrihydrite in the soil + ^{57}Fh^{13}GluC spectra was consistently smaller than that of the same feature in the ^{57}Fh + soil spectra (47.3 T vs. 48.1 T at 5 K). Narrower mean hyperfine fields have been previously reported for similar ferrihydrite-organic matter coprecipitates,[12,17-19] as the presence of organic matter is thought to interfere with crystal growth, leading to smaller ferrihydrite crystals and more distorted Fe octahedra.[20-22] To further assess the crystallinity of ferrihydrite in the ^{57}Fh and ^{57}Fh^{13}GluC (co-)precipitates, we additionally collected spectra at intermediate temperatures (Figure S8). Both the soil + ^{57}Fh and soil + ^{57}Fh^{13}GluC spectra are mostly ordered at 45 K, suggesting that the blocking temperatures of the (co-)precipitates was between 45 K and 77 K, in agreement with values in literature for ferrihydrite.[11] However, a direct comparison of the samples at lower temperatures (e.g., 35 K) revealed that the soil + ^{57}Fh spectra more closely resembled a sextet (as opposed to a collapsed feature or a doublet) than the soil + ^{57}Fh^{13}GluC sample, indicating that the soil + ^{57}Fh was more magnetically ordered, indicative of a higher degree of crystallinity or stronger inter-particle interactions. Lower ordering temperatures have been previously reported for Fe(III)(oxyhydr)oxides formed in the presence of organic carbon (e.g., refs. [12,20,21]) and is in agreement with spectroscopic studies showing that coprecipitation with organic carbon results in increased structural distortion and changes in the local coordination environments.[23-26] Based on these analyses, we conclude that the ^{57}Fe-labelled ferrihydrite in ^{57}Fh was slightly more crystalline than that in the ^{57}Fh^{13}GluC coprecipitate.

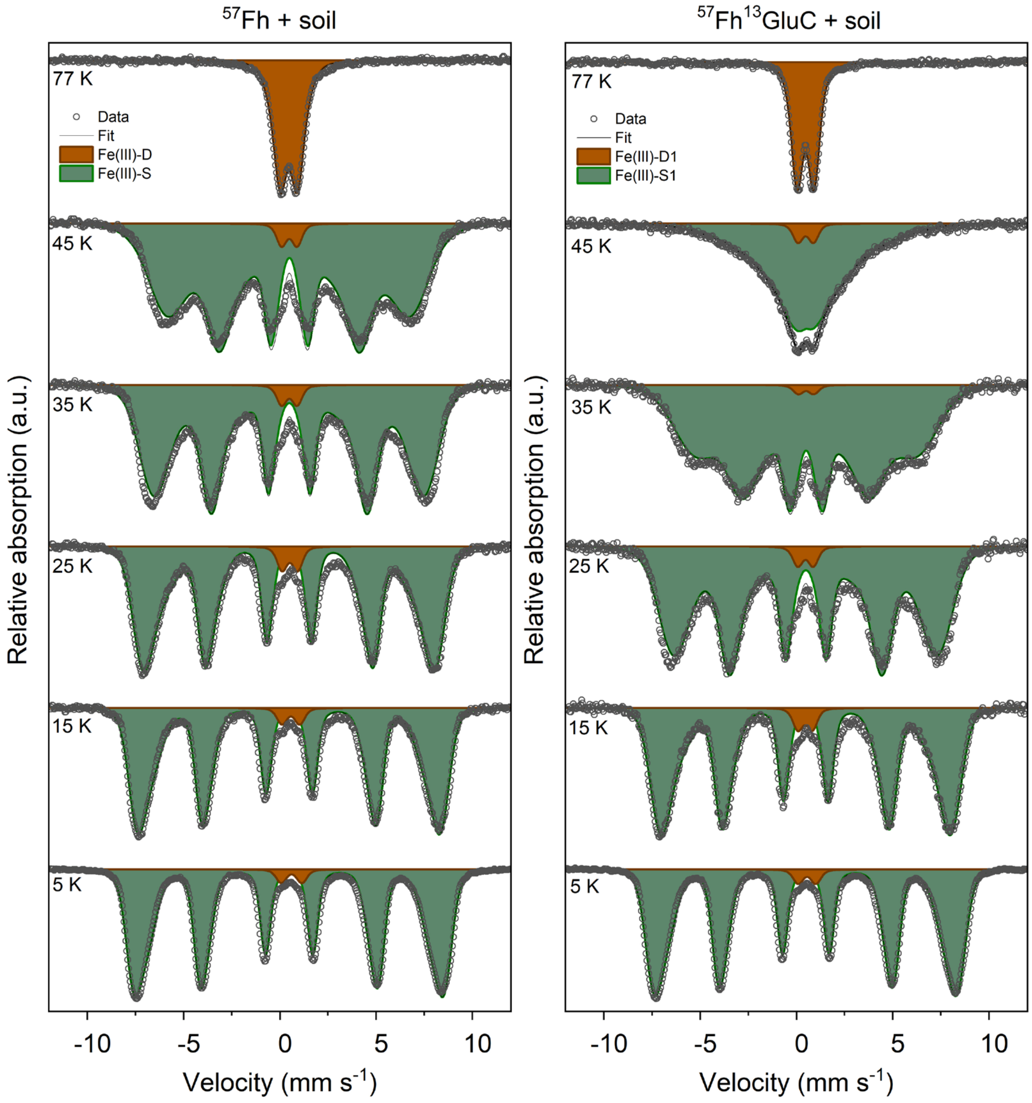

Figure S9. Temperature resolved Mössbauer spectra of the unreacted soil + ^{57}Fh and soil + ^{57}Fh^{13}GluC mixtures, collected at 77, 45, 35, 25, 15, and 5 K. Fit parameters are detailed in Table S8.

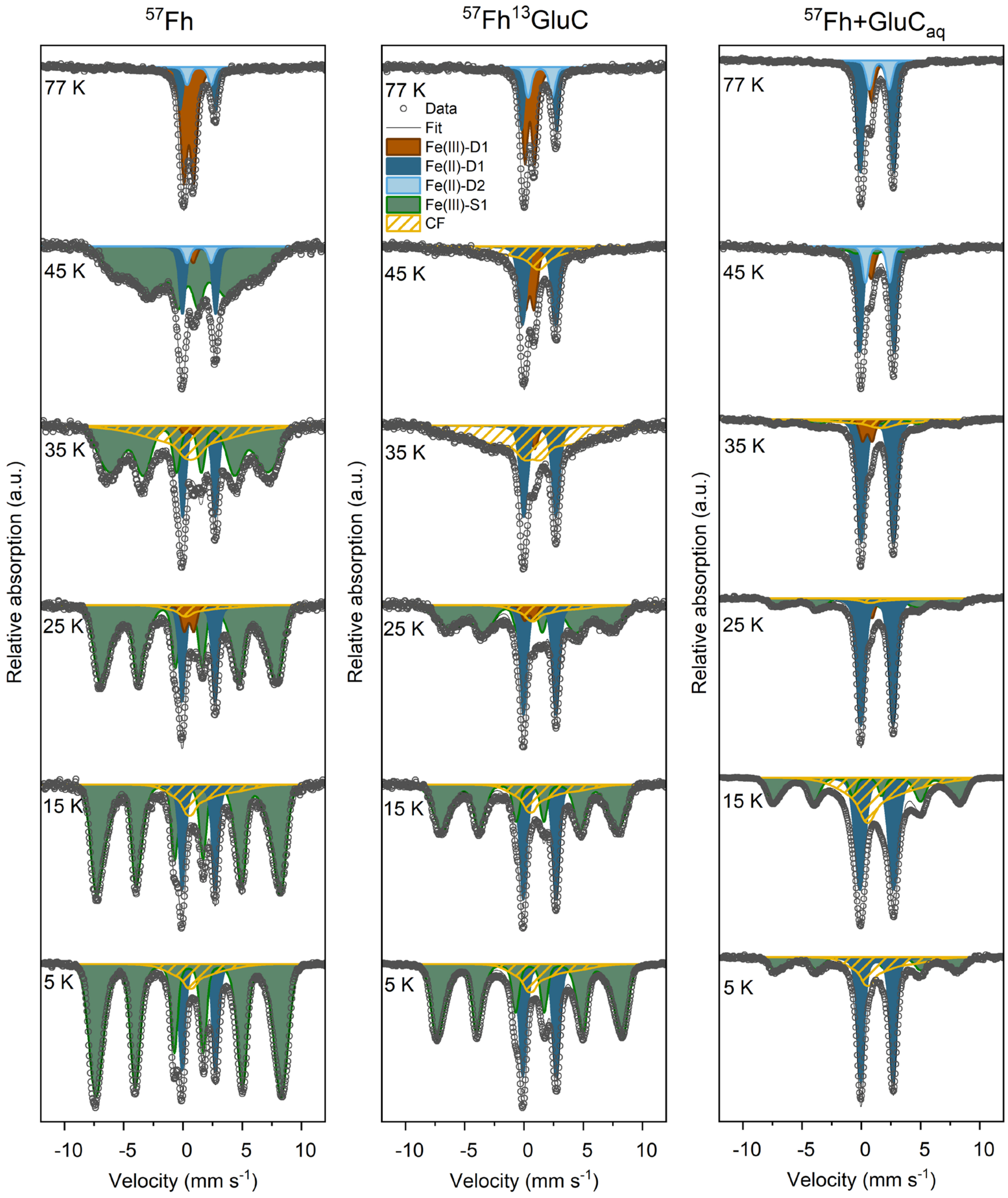

Figure S10. Temperature resolved Mössbauer spectra of the 6-week incubated samples collected at 77, 45, 35, 25, 15, and 5 K. Fit parameters are detailed in Table S9.

Table S7. Mossbauer fitting parameters of initial unreacted soil and the (co-)precipitates.

Sample	Temp.	Fe phase	Interpretation	Population (%)	CS[a] (mm s^{-1})	QS or ε[b] (mm s^{-1})	<|H|>[c] (T)	σ[d] (mm s^{-1}) or (T)	Reduced[e] χ^2
Unreacted Hestur_GA 60-72 soil	77 K	Fe(III)-D1	Fe(III) (oxyhydr)oxide paramagnetic at 77 K	65	0.49	0.72	-	0.3	3.93
		Fe(II)-D1	Paramagnetic Fe(II)	17	1.15	2.44	-	0.5	
		Fe(III)-S1	Fe(III) in Goethite	18	0.48	-0.12	47.7	3.7	
	5 K	Fe(III)-D1	Possibly Fe-OM or Fe-Si	13	0.48	0.75	-	0.4	2.59
		Fe(II)-D1	Paramagnetic Fe(II)	5	1.20	2.27	-	0.4	
		Fe(III)-S1	Fe(III) in Goethite and Ferrihydrite	27	0.49	-0.11	49.4	1.7	
		Fe(III)-S2	Likely ferrihydrite coprecipitated with carbon	32	0.50	0.00*	45.3	3.9	
		CF	Ordered Fe(II) phase + Fe(III) (oxyhydr)oxide near ordering temperature	23	0.73	0.00*	17.9	12.1	
^{57}Fh	77 K	Fe(III)-D	Ferrihydrite	100	0.45	1.33	-	1.1	1.15
		component 1		54	0.45	0.92	-	0.5	
		component 2		46	0.45	1.00*	-	2.0*	
	4 K	Fe(III)-S	Ferrihydrite	100	0.47	0.00	48.5	3.1	12.82
^{57}Fh^{13}GluC	77 K	Fe(III)-D	Ferrihydrite	100	0.46	0.96	-	0.6	1.04
		component 1		75	0.46	0.89	-	0.4	
		component 2		25	0.46	1.00*	-	1.0*	
	4 K	Fe(III)-S	Ferrihydrite	100	0.48	0.00	47.8	3.1	17.33

[a]Center shift with respect to α-^{57}Fe^{0}. [b]Quadrupole splitting, QS (for doublets) or quadrupole shift, ε (for sextets). [c]Mean hyperfine field. [d]Standard deviation of QS (doublets) or H (sextet). [e]Goodness of fit. Phases marked in italics are components of the previous phase. Note that the percentage of components always sums to 100% but refers to the percentage of the previous phase. *Indicates values that were fixed during the fitting process. Abbreviations: Fh = ferrihydrite.

Table S8. Mossbauer fitting parameters of unreacted (co-)precipitate + soil mixtures.

Sample		Temp.	Fe phase	Interpretation	Population (%)	CS[a] (mm s^{-1})	QS or ε[b] (mm s^{-1})	<|H|>[c] (T)	σ[d] (mm s^{-1}) or (T)	Reduced[e] χ^2
^{57}Fh + soil	Initial sample	77 K	Fe(III)-D	Ferrihydrite + Paramagnetic Fe(III)	100	0.46	1.06	-	0.7	1.42
			component 1		40		0.90	-	0.4	
			component 2		60		1*	-	1.0*	
		45 K	Fe(III)-D1	-	3	0.49	0.81	-	0.4	8.94
			Fe(III)-S1	-	97	0.47	-0.02	34.6	10.4	
		35 K	Fe(III)-D1	-	3	0.49	0.81	-	0.4	7.56
			Fe(III)-S1	-	97	0.48	-0.01	39.9	9.0	
		25 K	Fe(III)-D1	-	4	0.51	0.81	-	0.4	3.88
			Fe(III)-S1	-	96	0.48	-0.01	44.8	4.7	
		15 K	Fe(III)-D1	-	3	0.57	0.92	-	0.4	3.71
			Fe(III)-S1	-	97	0.48	0.00	47.0	3.5	
		5 K	Fe(III)-D1	Paramagnetic Fe(III)	2	0.60	1.07	-	0.4	37
			Fe(III)-S1	Ferrihydrite	98	0.48	0.00*	48.1	3.2	
^{57}Fh^{13}GluC + soil	Initial sample	77 K	Fe(III)-D1	Ferrihydrite + Paramagnetic Fe(III)	100	0.46	0.95	-	0.6	1.04
			component 1		75		0.87	-	0.4	
			component 2		25		1.00*	-	1.0*	
		45 K	Fe(III)-D1	-	5	0.48	0.81	-	0.4	0.54
			Fe(III)-S1	-	95	0.53	0.05	16.0	10.8	
		35 K	Fe(III)-D1	-	1	0.46	0.81	-	0.4	2.01
			Fe(III)-S1	-	99	0.48	-0.01	30.3	11.8	
		25 K	Fe(III)-D1	-	2	0.47		0.8	0.4	2.50
			Fe(III)-S1	-	98	0.48	-0.01	38.5	9.6	
		15 K	Fe(III)-D1	-	3	0.45	0.75	-	0.4	2.02
			Fe(III)-S1	-	97	0.48	0.00	45.0	4.3	
		5 K	Fe(III)-D1	Paramagnetic Fe(III)	3	0.55	0.92	-	0.4	15.16
			Fe(III)-S1	Ferrihydrite	97	0.48	-0.01*	47.3	3.2	

[a]Center shift with respect to α-^{57}Fe^0. [b]Quadrupole splitting, QS (for doublets) or quadrupole shift, ε (for sextets). [c]Mean hyperfine field. [d]Standard deviation of QS (doublets) or H (sextet). [e]Goodness of fit. Phases marked in italics are components of the previous phase. Note that the percentage of components always sums to 100% but refers to the percentage of the previous phase. *Indicates values that were fixed during the fitting process. Abbreviations: Fh = ferrihydrite.

Table S9. Mossbauer fitting parameters of 2- and 6-week incubated samples.

Sample		Temp.	Fe phase	Interpretation	Population (%)	CS[a] (mm s^{-1})	QS or ε[b] (mm s^{-1})	<|H|>[c] (T)	σ[d] (mm s^{-1}) or (T)	Reduced χ² [e]
^{57}Fh + soil	2-week incubated	77 K	Fe(III)-D1	Paramagnetic Fe(III)	92	0.47	0.98	-	0.6	0.63
			component 1		61		0.85	-	0.4	
			component 2		39		1.00*	-	1.0*	
			Fe(II)-D1	Paramagnetic Fe(II)	8	1.20	2.96	-	0.5*	
	6-week incubated	77 K	Fe(III)-D1	Paramagnetic Fe(III)	69	0.49	0.95	-	0.6	1.59
			component 1		63		0.82	-	0.4*	
			component 2		37		1.00*	-	1.0*	
			Fe(II)-D1	Paramagnetic Fe(II)	21	1.25	3.01	-	0.3	
			Fe(II)-D2	Paramagnetic Fe(II)	10	1.36*	2.04*	-	0.4*	
		45 K	Fe(III)-D1	-	3	0.41	0.93	-	0.4	0.82
			Fe(II)-D1	-	14	1.35	2.80	-	0.4*	
			Fe(II)-D2	-	3	1.36*	2.04*	-	0.4*	
			Fe(III)-S1 + CF	-	80	0.44	0.00	29.5	12.8	
		35 K	Fe(III)-D1	-	1	0.50	0.81	-	0.4	2.19
			Fe(II)-D1	-	17	1.34	2.75	-	0.4	
			Fe(III)-S1	-	58	0.50	0.00	41.2	6.0	
			CF	-	24	0.64	0.00	22.2	15.5	
		25 K	Fe(III)-D1	-	4	0.50	0.81	-	0.4	2.60
			Fe(II)-D1	-	17	1.31	2.77	-	0.4	
			Fe(III)-S1	-	74	0.47	-0.01	43.7	5.2	
			CF	-	5	1.20	1.00*	11.7*	8.0	

Table S9 continued. Mossbauer fitting parameters of 2- and 6-week incubated samples.

Sample		Temp.	Fe phase	Interpretation	Population (%)	CS[a] (mm s^{-1})	QS or ε[b] (mm s^{-1})	<|H|>[c] (T)	σ[d] (mm s^{-1}) or (T)	Reduced χ² [e]
^{57}Fh + soil	6-week incubated	**15 K**	Fe(II)-D1	-	16	1.32	2.79	-	0.4	1.49
			Fe(III)-S1	-	73	0.48	-0.01*	46.6	3.7	
			CF	-	11	1.51	1.00*	11.7*	8.0	
		**5 K**	Fe(II)-D1	Paramagnetic Fe(II)	15	1.33	2.82	-	0.4	14.1
			Fe(III)-S1	Fh like	78	0.47	-0.01*	48.0	3.5	
			CF	Ordered Fe(II) phase + Fe(III) (oxyhydr)oxide near ordering temperature	8	1.09	0.47	11.7*	8.0	
^{57}Fh^{13}GluC + soil	2-week incubated	**77 K**	Fe(III)-D1	Paramagnetic Fe(III)	82	0.48	0.96	-	0.6	0.93
			component 1		67		0.85	-	0.4	
			component 2		33		1.00*	-	1.0*	
			Fe(II)-D1	Paramagnetic Fe(II)	18	1.23	2.86	-	0.6	
	6-week incubated	**77 K**	Fe(III)-D1	Paramagnetic Fe(III)	52	0.49	0.92	-	0.5	1.93
			component 1		71		0.81	-	0.3	
			component 2		29		1.00*	-	1.0*	
			Fe(II)-D1	Paramagnetic Fe(II)	31	1.26	2.98	-	0.3	
			Fe(II)-D2	Paramagnetic Fe(II)	17	1.36*	2.04*	-	0.4*	
		**45 K**	Fe(III)-D1	-	28	0.48	0.82	-	0.5	1.19
			Fe(II)-D1	-	43	1.27	2.81	-	0.6	
			CF	-	29	0.46	-0.79	16.6	12.3	
		**35 K**	Fe(III)-D1	-	8	0.34	1.15	-	0.5	1.00
			Fe(II)-D1	-	33	1.33	2.70	-	0.5	
			CF	-	60	0.74	0.00*	27.9	17.4	

Table S9 continued. Mossbauer fitting parameters of 2- and 6-week incubated samples.

Sample		Temp.	Fe phase	Interpretation	Population (%)	CS[a] (mm s^{-1})	QS or ε[b] (mm s^{-1})	$\langle \lvert H \rvert \rangle$[c] (T)	σ[d] (mm s^{-1}) or (T)	Reduced χ^2 [e]
$^{57}Fh^{13}GluC$ + soil	6-week incubated	25 K	Fe(III)-D1	-	4	0.44	0.81	-	0.4	1.94
			Fe(II)-D1	-	32	1.32	2.73	-	0.5	
			Fe(III)-S1	-	53	0.49	0.00*	40.9	7.2	
			CF	-	10	1.69	1.00*	11.7*	8.0	
		15 K	Fe(II)-D1	-	29	1.31	2.76	-	0.5	2.65
			Fe(III)-S1	-	56	0.48	-0.02	45.7	4.3	
			CF	-	15	1.52	1.00*	11.7*	8.0	
		5 K	Fe(II)-D1	Paramagnetic Fe(II)	25	1.31	2.82	-	0.5	11.95
			Fe(III)-S1	Ferrihydrite like	62	0.47	-0.01*	47.1	3.7	
			CF	Ordered Fe(II) phase + Fe(III) (oxyhydr)oxide near ordering temperature	13	1.50	1.00*	11.7*	8.0	
$^{57}Fh+GluC_{aq}$ + soil	2-week incubated	77 K	Fe(III)-D1	Paramagnetic Fe(III)	79	0.51	0.92	-	0.6	2.25
			component 1		63		0.76	-	0.4	
			component 2		37		1.00*	-	1.0*	
			Fe(II)-D1	Paramagnetic Fe(II)	21	1.31	3.14	-	0.3	
	6-week incubated	77 K	Fe(III)-D1	Paramagnetic Fe(III)	20	0.48	0.91	-	0.5	2.61
			component 1		70	0.48	0.80	-	0.3	
			component 2		30	0.48	1.00*	-	1.0*	
			Fe(II)-D1	Paramagnetic Fe(II)	65	1.31	2.81	-	0.5	
			Fe(II)-D2	Paramagnetic Fe(II)	15	1.50	1.66	-	0.4	

Table S9 continued. Mossbauer fitting parameters of 2- and 6-week incubated samples.

Sample		Temp.	Fe phase	Interpretation	Population (%)	CS[a] (mm s^{-1})	QS or ε[b] (mm s^{-1})	<|H|>[c] (T)	σ[d] (mm s^{-1}) or (T)	Reduced χ^2 [e]
^{57}Fh+GluC$_{aq}$ + soil	6-week incubated	45 K	Fe(III)-D1	-	15	0.49	0.82	-	0.5	2.47
			Fe(II)-D1	-	51	1.30	2.89	-	0.4	
			Fe(II)-D2	-	15	1.36	2.04	-	0.4	
			Fe(III)-S1	-	20	0.43	0.00*	29.5	12.8	
		35 K	Fe(III)-D1	-	9	0.50	0.81	-	0.4	6.06
			Fe(II)-D1	-	65	1.34	2.75	-	0.6	
			Fe(III)-S1	-	13	0.60	0.00*	44.9	6.0	
			CF	-	14	1.63	0.00*	22.2	15.5	
		25 K	Fe(III)-D1	-	7	0.50	0.81	-	0.4	6.41
			Fe(II)-D1	-	64	1.30	2.73	-	0.6	
			Fe(III)-S1	-	25	0.49	0.00	44.0	5.6	
			CF	-	4	1.69	1.00*	11.7	8.0	
		15 K	Fe(II)-D1	-	50	1.31	2.74	-	0.5	3.17
			Fe(III)-S1	-	26	0.57	-0.08	46.9	4.3	
			CF	-	24	1.52	1.00*	11.7	8.0	
		5 K	Fe(II)-D1	Paramagnetic Fe(II)	40	1.29	2.84	-	0.7	76.39
			Fe(III)-S1	Ferrihydrite like	29	0.47	-0.01*	47.6	4.0	
			CF	Ordered Fe(II) phase + Fe(III) (oxyhydr)oxide near ordering temperature	31	1.50	1.00*	15.8	11.7	

[a]Center shift with respect to α-^{57}Fe^{0}. [b]Quadrupole splitting, QS (for doublets) or quadrupole shift, ε (for sextets). [c]Mean hyperfine field. [d]Standard deviation of QS (doublets) or H (sextet). [e]Goodness of fit. Phases marked in italics are components of the previous phase. Note that the percentage of components always sums to 100% but refers to the percentage of the previous phase. *Indicates values that were fixed during the fitting process. Abbreviations: Fh = ferrihydrite.

8. References

(1) ThomasArrigo, L. K.; Vontobel, S.; Notini, L.; Nydegger, T. Coprecipitation with ferrihydrite inhibits mineralization of glucuronic acid in an anoxic soil. *Environ. Sci. Technol.* **2023**, *57* (25), 9204-9213.

(2) WRB, I. W. G. World Reference Base for Soil Resources 2014. International soil classification system for naming soils and creating legends for soil maps. FAO, Rome. **2014**.

(3) Taylor, P. D. P.; Maeck, R.; Debievre, P. Determination of the absolute isotopic composition and atomic weight of a reference sample of natural iron. *Int. J. Mass Spectrom.* **1992**, *121*, 111-125.

(4) De Laeter, J. R.; Böhlke, J. K.; De Bièvre, P.; Hidaka, H.; Peiser, H. S.; Rosman, K. J. R.; Taylor, P. D. P. Atomic weights of the elements: Review 2000 - (IUPAC technical report). *Pure Appl. Chem.* **2003**, *75* (6), 683-800.

(5) Ravel, B.; Newville, M. ATHENA, ARTEMIS, HEPHAESTUS: data analysis for X-ray absorption spectroscopy using IFEFFIT. *J. Synchrotron Radiat.* **2005**, *12*, 537-541.

(6) ThomasArrigo, L. K.; Kretzschmar, R. Iron speciation changes and mobilization of colloids during redox cycling in Fe-rich, Icelandic peat soils. *Geoderma* **2022**, *428*, 116217.

(7) Loeppert, R. H.; Inskeep, W. P. Iron. In *Methods of Soil Analysis, Part 3. Chemical Methods*, Sparks, D. L., Page, A. L., Helmke, P. A., Loeppert, R. H., Soltanpour, P. N., Tabatabai, M. A., Johnston, C. T., Sumner, M. E. Eds.; Soil Science Society of America, 1996; pp 639-644.

(8) Fadrus, H.; Malý, J. Supression of iron(III) interference in determination of iron(II) in water by 1,10-phenanthroline method. *Analyst* **1975**, *100* (1193), 549-554.

(9) Tishchenko, V.; Meile, C.; Scherer, M. M.; Pasakarnis, T. S.; Thompson, A. Fe^{2+} catalyzed iron atom exchange and re-crystallization in a tropical soil. *Geochim. Cosmochim. Acta* **2015**, *148*, 191-202.

(10) McKeague, J. A. An evaluation of 0.1 *M* pyrophosphate and pyrophosphate-dithionite in comparison with oxalate as extractants of accumulation products in podzols and some other soils *Can. J. Soil Sci.* **1967**, *47* (2), 95-99.

(11) Byrne, J.; Kappeler, A. Mössbauer Spectroscopy. In *Analytical Geomicrobiology: A Handbook of Instrumental Techniques*, Kenney, J., Veeramani, H., Alessi, D. Eds.; Cambridge University Press, 2019; pp 314-338.

(12) Schwertmann, U.; Wagner, F.; Knicker, H. Ferrihydrite-humic associations: Magnetic hyperfine interactions. *Soil Sci. Soc. Am. J.* **2005**, *69*, 1009-1015.

(13) Chen, C. M.; Kukkadapu, R. K.; Lazareva, O.; Sparks, D. L. Solid-phase Fe speciation along the vertical redox gradients in floodplains using XAS and Mössbauer spectroscopies. *Environ. Sci. Technol.* **2017**, *51* (14), 7903-7912.

(14) Williams, A. G. B.; Scherer, M. M. Spectroscopic evidence for Fe(II)-Fe(III) electron transfer at the iron oxide-water interface. *Environ. Sci. Technol.* **2004**, *38*, 4782-4790.

(15) Chen, C. M.; Thompson, A. The influence of native soil organic matter and minerals on ferrous iron oxidation. *Geochim. Cosmochim. Acta* **2021**, *292*, 254-270.

(16) Byrne, J. M.; Kappler, A. A revised analysis of ferrihydrite at liquid helium temperature using Mossbauer spectroscopy. *Am. Miner.* **2022**, *107* (8), 1643-1651.

(17) ThomasArrigo, L. K.; Byrne, J.; Kappler, A.; Kretzschmar, R. Impact of organic matter on iron(II)-catalyzed mineral transformation in ferrihydrite-OM coprecipitates. *Environ. Sci. Technol.* **2018**, *52*, 12316-12326.

(18) ThomasArrigo, L. K.; Mikutta, C.; Byrne, J.; Kappler, A.; Kretzschmar, R. Iron(II)-catalyzed iron atom exchange and mineralogical changes in iron-rich organic freshwater flocs: An iron isotope tracer study. *Environ. Sci. Technol.* **2017**, *51*, 6897-6907.

(19) ThomasArrigo, L. K.; Mikutta, C.; Byrne, J.; Barmettler, K.; Kappler, A.; Kretzschmar, R. Iron and arsenic speciation and distribution in organic flocs from streambeds of an arsenic-enriched peatland. *Environ. Sci. Technol.* **2014**, *48*, 13218-13228.

(20) Chen, C.; Kukkadapu, R. K.; Sparks, D. L. Influence of coprecipitated organic matter on $Fe^{2+}{}_{(aq)}$-catalyzed transformation of ferrihydrite: Implications for carbon dynamics. *Environ. Sci. Technol.* **2015**, *49*, 10927-10936.
(21) Eusterhues, K.; Wagner, F. E.; Häusler, W.; Hanzlik, M.; Knicker, H.; Totsche, K. U.; Kögel-Knabner, I.; Schwertmann, U. Characterization of ferrihydrite-soil organic matter coprecipitates by X-ray diffraction and Mössbauer spectroscopy. *Environ. Sci. Technol.* **2008**, *42*, 7891-7897.
(22) Murad, E. The Mössbauer spectrum of 'well'-crystallized ferrihydrite. *J. Magn. Magn. Mater.* **1988**, *74*, 153-157.
(23) ThomasArrigo, L. K.; Kaegi, R.; Kretzschmar, R. Ferrihydrite growth and transformation in the presence of ferrous Fe and model organic ligands. *Environ. Sci. Technol.* **2019**, *53*, 13636-13647.
(24) Mikutta, C. X-ray absorption spectroscopy study on the effect of hydroxybenzoic acids on the formation and structure of ferrihydrite. *Geochim. Cosmochim. Acta* **2011**, *75*, 5122-5139.
(25) Mikutta, C.; Frommer, J.; Voegelin, A.; Kaegi, R.; Kretzschmar, R. Effect of citrate on the local Fe coordination in ferrihydrite, arsenate binding, and ternary arsenate complex formation. *Geochim. Cosmochim. Acta* **2010**, *74*, 5574-5592.
(26) Mikutta, C.; Mikutta, R.; Bonneville, S.; Wagner, F.; Voegelin, A.; Christl, I.; Kretzschmar, R. Synthetic coprecipitates of exopolysaccharides and ferrihydrite. Part 1: Characterization. *Geochim. Cosmochim. Acta* **2008**, *72*, 1111-1127.
